# Engineering of a novel optimized platform for sublingual delivery with novel characterization tools: *in vitro* evaluation and *in vivo* pharmacokinetics study in human

**DOI:** 10.1080/10717544.2017.1334719

**Published:** 2017-06-09

**Authors:** Nadia M. Morsi, Ghada A. Abdelbary, Ahmed H. Elshafeey, M. Abdallah Ahmed

**Affiliations:** Department Pharmaceutics and Industrial Pharmacy, Faculty of Pharmacy, Cairo University, Cairo, Egypt

**Keywords:** Phosphatidylinositol, complexosomes, sublingual platform, flush resistant, sodium alginate

## Abstract

The aim of this work was to develop a novel and more efficient platform for sublingual drug delivery using mosapride citrate (MSP) as a model drug. The engineering of this delivery system had two stages, the first stage was tuning of MSP physicochemical properties by complexation with pure phosphatidylcholine or phosphatidylinositol enriched soybean lecithin to form MSP-phospholipid complex (MSP-PLCP). Changes in physicochemical properties were assessed and the optimum MSP-PLCP formula was then used for formulation into a flushing resistant platform using two mucoadhesive polymers; sodium alginates and sodium carboxymethylcellulose at different concentrations. Design of experiment approach was used to characterize and optimize the formulated flushing resistant platform. The optimized formulation was then used in a comparative pharmacokinetics study with the market formulation in human volunteers. Results showed a marked change in MSP physicochemical properties of MSP-PLCP compared to MSP. Addition of mucoadhesive polymers to flushing resistant platform at an optimum concentration balanced between desired mucoadhesive properties and a reasonable drug release rate. The optimized formulation showed significantly a superior bioavailability in humans when compared to the market sublingual product. Finally, the novel developed sublingual flushing resistant platform offers a very promising and efficient tool to extend the use of sublingual route and widen its applications.

## 1. Introduction

Sublingual route is known to be one of the most effective routes for drug delivery with many advantages over the other routes. One of these advantages is direct absorption of drug into systemic circulation (Bayrak et al., 2011; Hearnden et al., 2012). Bypassing gastrointestinal tract enzymatic degradation and excellent accessibility are other advantages of sublingual route (Rathbone & Hadgraft, 1991). On the other hand, sublingual route has many problems, which limit its use. One of these problems is the involuntary swallowing of liquids into gastrointestinal tract and rapid drug elimination due to the flushing action of saliva. This concept is known as ‘saliva wash out’ (Patel et al., 2011). Because of this, the main application of the sublingual route is somewhat limited to the delivery of small molecules when rapid onset of action is desirable (e.g. nitroglycerin) (Harris & Robinson, 1992). The main application of the sublingual route is the delivery of small molecules (<75–100 Da) or molecules with the ideal lipophilic character (log *P*) for maximum absorption (4.2–5.5) (Harris & Robinson, 1992).

Upon designing a delivery system for sublingual absorption, one of the most important factors is the lipid nature of the designed molecule (Harris & Robinson, 1992). The drug ability to permeate into human oral mucosa is dependent on its lipid solubility, which is measured by its oil/water partition coefficient (Beckett & Moffat, 1969). Sublingual absorption is comparatively slow for the compounds with high molecular weight or low lipophilicity, while moderate lipophilicity drugs are well absorbed (De Boer et al., 1984). On the other hand, solubility of drugs with a very high partition coefficient is too low to achieve sufficient concentration in salivary fluids (De Boer et al., 1984).

In order to extend the use of sublingual route and widen its applications, drug maintenance in the sublingual area is an important obstacle that must be taken into consideration (De Boer et al., 1984). Drug maintenance in the sublingual area is dependent mainly upon the drug formulation and varies between subjects (De Boer et al., 1984). From formulation point of view, adhesion to the moist surface of mucosa is essential to resist the flushing action of saliva; hence, bioadhesive polymers are used during formulation (Bayrak et al., 2011). On the other hand, Drug concentrations can be sustained only for a relatively short period of time, probably in the order of only minutes (Patel et al., 2011). This point should be considered when formulating a mucoadhesive sublingual dosage form.

Drug–phospholipid interaction approach was employed to increase the drug lipid solubility. Recently, new phospholipid–drug interaction approaches emerged as a tool to adjust physicochemical properties of biologically active molecules with the ultimate goal to improve its bioavailability. During this study, one of these approaches was used. This approach depends on weak physical interaction by hydrogen bond formation and electrostatic interaction between drug molecule and phospholipids to form drug–phospholipid complex (Yanyu et al., 2006; Maiti et al., 2007; Yue et al., 2010; Yue et al., 2010; Cai et al., 2012; Guo et al., 2014; Singh et al., 2014). The prepared drug–phospholipid complex is then formulated into a flushing resistance platform with the addition of a mucoadhesive polymer. Upon salivary hydration, the formula undergoes initial mucoadhesion to the sublingual area followed by formation of flushing resistant mucoadhesive dispersion containing nano-sized drug–phospholipid complex vesicles or what can be named as complexosomes. These complexosomes have a moderate lipophilicity, which is ideal for sublingual absorption and possess mucoadhesive characteristics. During the study, novel characterization tools of the prepared flushing resistant platform were used.

For the application of this approach, mosapride citrate (MSP) was chosen as a model drug. MSP(PubChem CID: 119583) is a selective 5-HT5 agonist, which promotes upper gastrointestinal motility with low lipophilicity (Ruth et al., 1998; Ruth et al., 2003; Curran & Robinson, 2008; Patil et al., 2009). MSP is a prokinetic agent with no severe side effects and which can be safely used for further *in vivo* studies (Ruth et al., 1998). Dose of MSP is 5 mg and it has very low oral bioavailability of about 8% (Sakashita et al., 1993). MSP is available as a fast dissolving tablet, which can be used for *in vivo* comparison.

## 2. Materials and methods

### 2.1. Materials

Mosapride citrate dihydrate was kindly supplied by Marcyrl Company (Cairo, Egypt). l-α-Phosphatidylcholine from soybean type IV-S (SB1), l-α-phosphatidylcholine from soybean type II-S (SB2), sodium alginate and sodium carboxymethyl cellulose were purchased from Sigma-Aldrich (St. Louis, MO). Tetrahydrofuran, methanol, and *n*-octanol were purchased from Fisher Scientific UK Ltd. (Loughborough, England) sodium chloride powder, disodium hydrogen phosphate, potassium dihydrogen phosphate, orthophosphoric acid, glycine, and gelatin were purchased from El Nasr pharmaceutical company (Cairo, Egypt). Pearlitol 160C (Mannitol) was obtained from Roquette Group (Lille, France). Saccharin sodium and tutti frutti flavoring agent were purchased from Luna Group for Chemicals (Cairo, Egypt).

### 2.2. Preparation of MSP-phospholipid complexosomes (MSP-PLCP)

MSP-PLCP were prepared by employing the solvent-evaporation method (Yanyu et al., 2006; Maiti et al., 2007; Yue et al., 2010; Yue et al., 2010; Cai et al., 2012; Guo et al., 2014; Singh et al., 2014; Kassem et al., 2017; Jena et al., 2014; Cheng et al., 2015; Khurana et al., 2017). Briefly, weighed amounts of MSP and PLs were refluxed in a 100-ml rounded bottom flask containing tetrahydrofuran as reaction solvent at 60 °C for 2 h. Tetrahydrofuran was then evaporated off under vacuum at 40 °C. The dried residues were gathered and placed in desiccator overnight, then crushed in the mortar and sieved with a 100-mesh sieve. The composition of the prepared MSP-PLCP is listed in Table 1. Two different types of phospholipids were employed to investigate the influence of phospholipid composition on MSP-PLCP preparation. Two molar ratios of MSP: soybean lecithin (1:1 and 1:2) were prepared to evaluate effect of molar ratios on MSP-PLCP preparation.

**Table 1.** Composition of prepared MSP-PLCPs, percent yield, *n*-octanol/water partition coefficient (*P*), water solubility, *n*-octanol solubility, *in vitro* mucoadhesion time particles size, and PDI of MSP and MSP-PIPs.

Formula	Phospholipid type (PL)	Molar ratio (MSP:PL)	Yield (%) ± SD^a^	P ± SD^a^	Log P	Solubility in water (mg/ml) ±SD^a^	Solubility in *n*-octanol (mg/ml) ±SD^a^	*In vitro* flushing resistance time (s)±SD^a^	Particle size (z-average nm)±SD^a^	PDI ± SD^a^
MSP	–	–	–	5.74 ± 0.08	0.76	1.34 ± 0.13	1.88 ± 0.39	7.00 ± 1.41	–	–
CP 1	SB 1	1:1	60.79 ± 5.78	19.46 ± 1.98	1.29	0.71 ± 0.02	2.56 ± 0.09	62.50 ± 10.60	372.70 ± 21.78	0.36 ± 0.029
CP 2	SB 1	1:2	76.87 ± 1.37	8.95 ± 0.13	0.95	0.72 ± 0.05	1.74 ± 0.21	150.00 ± 14.14	246.25 ± 6.86	0.38 ± 0.017
CP 3	SB 2	1:1	90.90 ± 2.27	62.33 ± 2.62	1.79	0.68 ± 0.08	3.94 ± 0.55	26.50 ± 4.94	356.95 ± 47.45	0.27 ± 0.019
CP 4	SB 2	1:2	96.06 ± 0.187	49.21 ± 9.94	1.69	0.32 ± 0.01	4.23 ± 0.14	99.00 ± 21.21	268.80 ± 1.56	0.40 ± 0.006

^a^ All readings are average of three replicates.

### 2.3. *In vitro* characterization of MSP-PLCP

#### 2.3.1. Determination of the yield percent of MSP-PLCP

MSP interaction with PLs was determined by calculating the difference between total MSP and free MSP. To determine total MSP (a), weighed amount of prepared MSP-PLCP was dissolved in methanol and then measured spectrophotometrically at *λ*_max_ 308 nm. To determine free MSP (b), weighed amount of the prepared MSP-PLCP (equal to the weighed amount in the first step) was washed by 1% acetic acid in which MSP is freely soluble while PL forms a colloidal dispersion. The formed dispersion is then filtered through a double 0.22-μm membrane to remove any extracted colloidal PL. The obtained clear liquid containing free MSP dissolved in 1% acetic acid was then determined spectrophotometrically at *λ*_max_ 308 nm. The yield percent was calculated using the following equation:

(1) $$Yield\;percent=\frac{a-b}{a}\times 100,$$

where *a* is total MSP and *b* is free MSP.

#### 2.3.2. Determination of *n*-octanol/water partition coefficient (*P*) of MSP and MSP-PLCP

Determination of *n*-octanol/water partition coefficient of MSP and MSP-PLCP was carried out by the addition of weighed amount of MSP or MSP-PLCP to 10 ml water in sealed glass containers at 37 °C. Mixtures were then agitated for 24 h and centrifuged to remove excess residues (15 min, 4000 rpm). After removing excess residue, 10 ml *n*-octanol was then added and agitated for 24 h. For separation of the water phase and *n*-octanol phase, these mixtures were centrifuged at 4000 rpm for 15 min. The water phase and *n*-octanol phase were separately filtrated through a double 0.22 μm membrane to obtain a clear liquid. The aqueous concentration of MSP was measured spectrophotometrically at wave length 308 nm. The concentration of MSP in octanol was measured spectrophotometrically after dilution with methanol at wave length 308 nm. The partition coefficient was calculated from the following equation:

(2) $$P\;=\frac{C_{\text{o}}}{C_{\text{w}}},$$

where *C*_o_ was the concentration of MSP in *n*-octanol; *C*_w_ was the concentration of MSP in water.

#### 2.3.3. Solubility studies in water and *n*-octanol

Excess amounts of MSP or MSP-PLCP were added to 5 ml distilled water (pH 6.9) or *n*-octanol in a sealed glass container at 37 °C. The liquids were agitated for 24 h and then centrifuged to remove excessive MSP and MSP-PLCP. The obtained solution was filtrated through a double 0.22-μm membrane to obtain a clear solution. The absorbance of each system was then recorded in ultraviolet–visible spectrophotometer to calculate saturated solubility.

#### 2.3.4. Measurement of *in vitro* simulated sublingual flushing resistance time (SFRT)

From the formulation point of view, there is a need of adhesion to the moist surface of mucosa to resist the flushing action of saliva (Bayrak et al., 2011). Hence, it was important to evaluate the mucoadhesive characters of prepared MSP-PLCP. A method introduced by Nakamura et al. with slight modification was employed (Nakamura et al., 1996). A hot solution of agar/mucin, 1%/2%, w/w, in pH 6.8 buffer, was prepared and poured while hot in a Petri dish with 6 cm diameter and then left to gel at 4–8 °C for 3 h (Bertram & Bodmeier, 2006). MSP powder or MSP-PLCP were attached to the adhesive side of double-sided adhesive tape (square part 1 cm ×1 cm) so it covered the whole adhesive area. The loaded adhesive tape was then placed in the center of the petri dish so that the solid particles face the agar/mucin surface. A weight of 20 g was then placed over the adhesive tape for one minute to assure intimate contact between solid and agar/mucin surface. The petri dish was then attached to disintegration test apparatus, and moved up and down in pH 6.8 buffer at 37 °C as shown in Figure S1 in Supplementary material. The sample on the plate was immersed into the solution at the lowest point, and was out of the solution at the highest point. The residence time of the samples on the plate was recorded in seconds using a stopwatch.

#### 2.3.5. Particle size (PS) analysis of MSP-PLCP

MSP-PLCP were properly dispersed in distilled water to form a diluted dispersion with suitable scattering intensity. The mean PS and PDI were then determined by Malvern Zetasizer at 25 °C (Malvern Instrument Ltd., Worcestershire, UK).

#### 2.3.6. Transmission electron microscopy

Morphology of dispersed MSP-PLCP was examined using TEM (Jeol-200 CX, Joel, Tokyo, Japan). A drop of the dispersed MSP-PLCP was placed in the form of a thin film on a carbon-coated copper grid and then viewed and photographed under TEM.

#### 2.3.7. Fourier-transform infrared spectroscopy (FT-IR)

FT-IR spectra between 4000 and 500 cm^−1^ of the drug, drug–phospholipid powder mixtures and MSP-PLCP were determined using the potassium bromide (KBr) disc technique (FTIR-8400 S, Shimadzu, Kyoto, Japan).

#### 2.3.8. Differential scanning calorimetry (DSC)

Samples (3–4 mg) were placed in aluminum pan and heated at a rate of 10 °C/min, with indium in the reference pan, in an atmosphere of nitrogen to a temperature of 400 °C (DSC-50, Shimadzu). The DSC studies were performed for drug, drug–phospholipid powder mixtures and MSP-PLCP.

#### 2.3.9. Powder X-ray diffraction (XRD)

X-ray diffraction experiments were performed in a Scintag (Scintag, Inc., Cupertino, CA) Xray diffractometer using Cu Kα radiation with a nickel filter, a voltage of 45 kV, and a current of 40 mA. Diffraction patterns of drug, drug–phospholipid powder mixtures and MSP-PLCP were obtained.

#### 2.3.10. Statistical analysis

A 2^2^ full factorial experimental design was used to investigate the effect of phospholipid type and molar ratio on the percent yield, *n*-octanol/water partition coefficient (*P*), solubility in water, solubility in *n*-octanol, *in vitro* SFRT and the average PS (Z-average) of MSP-PLCPs using Design Expert^®^ (version 10.0.3, Stat-Ease, Minneapolis, MN). After analysis of the factorial design, desirability factor was determined for the choice of optimum MSP-PLCP formula for further studies. The criteria for the optimization were set at the highest percent yield, partition coefficient and *in vitro* mucoadhesion time and the lowest PS.

### 2.4. Preparation and characterization of sublingual flushing resistant platform (SFRP) containing the optimum MSP-PLCP formula

#### 2.4.1. Preparation of SFRP containing the optimum MSP-PLCP formula

The detailed composition of the prepared flushing resistant platform (SFRP) containing the optimum MSP-PLCP formula is presented in Table 2. Gelatin (2% w/w) was used a matrix former, a sugar alcohol (1% w/w mannitol) was used as a cryoprotectant and 0.1% w/w glycine was used as a collapse protectant. Sodium alginate (Na alginate), and sodium carboxymethylcellulose (Na CMC) were used as bioadhesive polymers in different concentrations. Gelatin was first dissolved in distilled water at about 40 °C. Mannitol, glycine, bioadhesive polymer, 0.5% w/w saccharin sodium, and 0.25% w/w tutti fruitti flavor were then added to the gelatin solution. Accurately weighed amount of MSP-PLCP in a dose equivalent to 5 mg MSP per 200 mg was then dispersed in the prepared aqueous gelatin solution using a magnetic stirrer. Two hundred milligrams of the resulting dispersion was then poured into each pocket of a PVC blister pack with a diameter of 12 mm and a depth of 4 mm resulting in a dose equivalent to 5 mg MSP per SFRP. The tablet blister packs were then transferred to a freezer at −22 °C and kept or 24 h. The frozen SFRPs were then placed for 24 h in Savant Novalyphe-NL 500 freeze dryer with a condenser temperature of −45 °^ο^C and a pressure of 7 × 10^−2^ mbar (Savant Instruments, NY). The prepared SFRPs were kept in tightly closed containers in desiccators over calcium chloride (0% relative humidity) at room temperature until further use.

**Table 2.** Composition, disintegration time, wetting time, USP Q3, USP Q10, SSDT Q2, SSDT Q10, mucoadhesion time, and bioadhesion force of the prepared tablets.

Run	Polymer concentration (%)	Polymer type	Disintegration time (s)	Wetting time (s)	USP Q3	USP Q10 (% released)	SSDT Q2 (% released)	SSDT Q10 (% released)	SFRT (s)	SFRF (dyne/cm^2^)
1	0	Na alginate	22	5	100	100	10.125	100	11	510.5
2	2	Na CMC	9	180	60.86	100	2.67	23.6	74	970.2
3	2	Na alginate	13	180	53.45	92.73	2.43	48.2	1836	1195.92
4	2	Na alginate	15	180	50.48	94.04	2.19	49.1	1860	1207.8
5	0	Na CMC	27	5	100	100	11.2	100	11	515.89
6	0	Na CMC	22	6	100	100	9.9	100	14	524.87
7	1	Na CMC	3	4	100	100	16.3279	66.1	61	863.28
8	2	Na CMC	11	180	63.8	98.6	2.31	26.1	88	926.64
9	1	Na CMC	5	7	100	100	17.0526	68.4	65	582.12
10	0	Na alginate	25	6	100	100	10.935	100	13	520.4

#### 2.4.2. *In vitro* disintegration and wetting time

Disintegration times for six SFRPs in simulated saliva at 37 ± 0.5 °C were determined using a USP disintegration tester. A digital stopwatch was used to measure the disintegration time to the nearest second. According to the European pharmacopeia, orodispersible tablets should disintegrate within 3 min when examined by the test for disintegration. Therefore, for SFRPs which disintegrate after 3 min, the disintegration time was taken as >3 min. For determination of wetting time, SFRPs were carefully placed in the center of the Petri dish of 10 cm diameter containing 10 ml of eosin dye solution in distilled water. The wetting time was taken as the time required for colored solution to reach the upper surface of the SFRP (Schiermeier & Schmidt, 2002).

#### 2.4.3. Determination of *in vitro* simulated sublingual dissolution time (SSDT) using a novel developed method

The sublingual route has special anatomical and physiological conditions when compared to other parts of GIT. Dosage forms in sublingual cavity are exposed to a small volume of saliva. In adults, normal resting salivary flow rate ranges from 0.25 to 0.35 ml/min, while stimulated flow rate is about 1.5 ml/min (De Almeida et al., 2008). The available pharmacopeias’ dissolution methods do not simulate these special conditions for the sublingual cavity.

**Figure 1.**
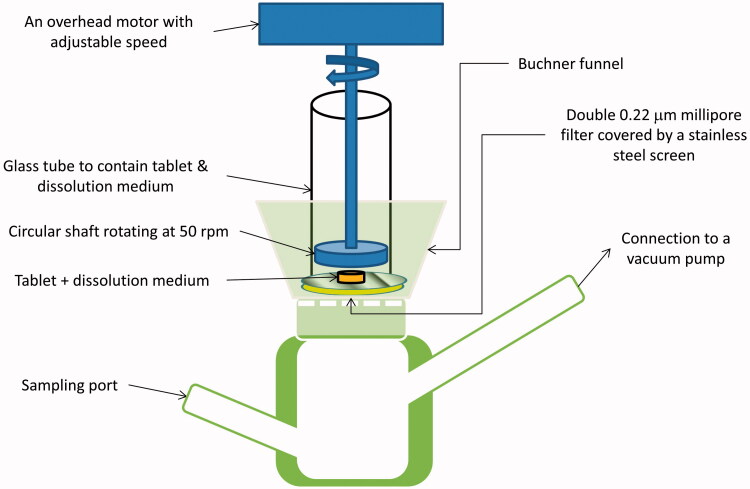
Schematic diagram for the new developed *in vitro* SSDT apparatus.

US Pharmacopeia (USP) dissolution method recommended for isosorbide dinitrate sublingual tablets uses paddle apparatus, 900 ml of water as dissolution medium and rotation speed of 50 rpm. These conditions are far from being the actual conditions in the sublingual cavity. A newly developed *in vitro* simulated sublingual dissolution method is needed to evaluate sublingual dosage forms. This method should be able to detect any minor changes in sublingual dosage form dissolution, which is related to formulation changes. Due to the limited volume of saliva and short residence time, minor changes in formulations and additives can greatly affect the rate and extent of sublingual absorption (Rachid et al., 2011). In this study, a new custom-made dissolution apparatus was used to evaluate the prepared SFRPs. The newly developed *in vitro* SSDT studies were performed for all SFRPs and for the commercial product Fluxopride^®^. Figure 1 represents a schematic diagram for the new developed *in vitro* SSDT apparatus.

As shown in Figure 1, the apparatus consists of four main parts. The lower part consists of a custom-made glass container with two ports. The lower left-hand port is a sampling port, while the upper right hand port is connected to a vacuum pump. A Buchner funnel is connected firmly above the lower glass part. Two 0.22-μm millipore filters covered by a stainless-steel screen are fitted above Buchner funnel surface, covering the holes in the funnel. A wide glass tube is fitted above the Buchner funnel. The purpose of this glass tube is to contain the tested SFRP and dissolution medium. The last part is an overhead motor with adjustable speed connected to rotating circular shaft.

The tested SFRP was placed into the glass tube, above the stainless-steel screen. The space between the circular shaft and the screen must be adjusted so that the SFRP can move freely. Three milliliters of dissolution medium were added (distilled water), and then the shaft was allowed to rotate at 50 rpm for 2 min. This rotation mimics the real sublingual condition, where the tongue natural movement accelerates the disintegration of the SFRPs. After 2 min, the vacuum pump was operated with vacuum so that the dissolution medium with dissolved drug was sucked into the lower glass part. After suction of the complete volume of dissolution medium, the vacuum pump was turned off and the sample was withdrawn from the sampling port and analyzed spectrophotometrically at *λ*_max_ 308 nm. Another 3 ml of dissolution medium were added and the previous steps were repeated for another four times with 2 min interval. The first sample represented the amount dissolved during the first 2 min and the second sample represented the amount dissolved from 2 to 4 min and so on. The cumulative amount dissolved at each time was calculated and the percent dissolved is then plotted against time.

#### 2.4.4. Measurement of *in vitro* SFRT (sublingual flushing resistance time)

*In vitro* SFRT was determined using the same method used for MSP-PLCP except that the double-sided adhesive tape with MSP-PLCP was replaced by the tested SFRP. This test evaluates the mucoadhesion properties of the whole SFRP before dispersion.

#### 2.4.5. Measurement of SFRF (sublingual flushing resistance force)

After administration of SFRP, the system will undergo fast initial disintegration followed by dispersion in the limited volume of saliva forming a flushing resistance platform. Therefore, for actual simulation of *in vivo* sublingual conditions, the bioadhesion force of SFRP after dispersion must be evaluated. The bioadhesion force was measured by a modified method of the viscosity test described by Hassan and Gallo (Hassan & Gallo, 1990).

Physical entanglements, conformational changes, and chemical interactions occur during the chain interpenetration of mucoadhesive polymers with mucin macromolecules results in changes in the rheological behavior of the two-macromolecular species. Subsequently, an increase in viscosity due to synergism in a mucin–polymer system is obtained. An evaluation of the resulting synergistic increase in viscosity can be determined by classical rotational viscometers at a certain shear rate (Schäfer-Korting, 2010).

Mucin dispersion (20% w/v) was prepared by gentle stirring of dried pork mucin with simulated saliva (pH 6.8) for 3 h at 25 °C. To get accurate results, all components’ concentrations must be kept constant during each viscosity measurement. In addition, for each viscosity measurement the volume must be constant. In our study, 6 ml was used for each viscosity measurement, and the viscosity was measured at 25 °C using LV Brookfiled (Brookfield Engineering Laboratories Inc, MA) viscometer at a rate of shear 15.84 s^−1^. To measure the viscosity of mucin alone, 5 ml of 20% w/v mucin dispersion was mixed with 1 ml simulated saliva and then the viscosity was measured (η_m_). To measure the viscosity of the dispersed SFRP, it was dispersed in 6 ml simulated saliva and then the viscosity was measured (η_d_). To measure the viscosity of mucin/dispersed SFRP system, SFRP was dispersed in 1 ml simulated saliva and then mixed with 5 ml of 20% w/v mucin dispersion for 15 min and then the viscosity was measured (η_t_). Accordingly, the mucin concentration measured every time was kept constant at 16.66% w/v. In addition, in each measurement the SFRP components were dispersed in a total volume of 6 ml.

The following equations were used to calculate the bioadhesion force (Hassan & Gallo, 1990):

(3) $$\eta_{\text{t}}=\eta_{\text{m}}+\eta_{\text{d}}+\eta_{\text{b}},$$

where η_t_ is the viscosity coefficient of the system, η_m_ and η_d_ are individual viscosity coefficients of mucin and dispersed SFRP, respectively. η_b_ is the viscosity component due to bioadhesion and can be obtained by rearranging equation (3):

(4) $$\eta_{\text{b}}=\eta_{\text{t}}-\eta_{\text{m}}-\eta_{\text{d}}.$$

Then the sublingual flushing resistance force (SFRF) resulting from bioadhesion was determined by:

(5) $$\text{SFRF }=\eta_{\text{b}}\sigma \text{,}$$

where η_b_ is the viscosity component due to bioadhesion obtained from Equation (4) and *σ* is the rate of shear per second.

#### 2.4.6. Experimental design

Design of experiments (DoE) is a planned approach to really understand cause-and-effect relationships (Anderson & Whitcomb, 2016). To perform a designed experiment, we must make changes to the input variables or factors and observe the changes in the output responses (Montgomery, 2008). A regression model is needed to establish a relationship between a response and a set of variables, which affect that response (Antony, 2014). Two of the most important reasons for fitting a regression models to data are interpretive purposes and using the model to predict the response for combinations of different variables at their optimum level, or what is called as optimization (Smith, 2005). The first step in development of a regression model is determination of regression coefficients (Antony, 2014). In general, the efficiency of regression coefficient estimator for a parameter increases as its variance becomes smaller, as larger estimator variance increases the uncertainty level about that estimator (Berger & Wong, 2009). For a set of parameter estimators, uncertainty can be expressed by the volume of a confidence ellipsoid, where the length of ellipsoid axes is related to each estimator variance (Berger & Wong, 2009). Hence, the smaller the volume of this confidence ellipsoid, the smaller the estimator variances and the more accurate the estimators. The determinant or D-optimality is a criterion for choosing design points that minimizes the product of the squared lengths of the axes of the ellipsoid, hence the volume of the confidence ellipsoid (Berger & Wong, 2009; Anderson & Whitcomb, 2016).

During this part of the study, D-optimal response surface experimental design was applied to investigate the effect of two independent variables including mucoadhesive polymer concentration (A) and the two-leveled categorical variable (mucoadhesive polymer type) (B) on the physicochemical properties of the prepared SFRPs. Design Expert software (version 10.0.3, Stat-Ease) was used for analysis and modeling of the responses. Based on the software calculations, a set of points consisted of 10 runs were selected as shown in Table 2. The software was also used for data statistical analysis and plotting of the response graphs. ANOVA test was used to evaluate the significance effect of the variables on the responses (*p* value <0.05). Second-order polynomial function was fitted to correlate the design variables and the responses. Multiple correlation coefficients (*R*^2^), adjusted *R*^2^ and predicted *R*^2^ were used to evaluate polynomial model equations. For optimization, criteria were set at the lowest disintegration and wetting time and the highest SSDT Q2, SSDT Q10, *in vitro* SFRT and SFRF. Finally, for model validation, five replicates of the optimized formula were prepared, the experimental responses were measured and then compared with the model predicted values. Morphology of optimum SFRP was also examined using TEM (Jeol-200 CX, Joel). A drop of the dispersed SFRP in 1  ml simulated saliva was placed in the form of a thin film on a carbon-coated copper grid and then viewed and photographed under TEM. The optimized formula was then used for further *in vivo* study.

### 2.5. Pharmacokinetic study in healthy volunteers

#### 2.5.1. Study design

Two-way crossover design with 1 week washout period was used. Randomly selected six human volunteers were divided into two groups. On the first day of the study phase one, the market reference product Fluxopride^®^ 5 mg tablet (Marcyrl) was administered to group I volunteers (*n* = 3) and the optimized test formula was administered to group II volunteers (*n* = 3) and vice versa in the study second phase. After fasting overnight, a control venous blood specimen was withdrawn from each volunteer on 0 h, then the specified regimen was administered sublingually for both the market reference the tested formula. Water or fluids were prohibited for 2 h after the dose and then standard meals were provided in normal time intervals. Blood samples were then collected at 0.25, 0.5, 0.75,1, 1.25, 1.5, 2, 2.5, 3, 4, 6, 8, and 10 h postdose.

#### 2.5.2. Subject selection

Six healthy human male volunteers with age range between 25 and 45 years were selected for the study. The volunteers had no history of drug or alcohol abuse and were free from chronic diseases. The details of the study were explained before starting the study and written consent was taken from all volunteers. The study was approved by the Faculty of Pharmacy Cairo University Research Ethics Committee, Cairo, Egypt (approval no. PI 1171).

#### 2.5.3. LC–MS/MS assay of MSP in human plasma

A validated LC–MS/MS method developed by Badawy et al. for the MSP detection in human plasma using itopride as an internal standard was utilized to estimate MSP content in plasma samples (Badawy et al., 2016). The collected heparinized blood samples were centrifuged at 3500 rpm for 10 min at 4 °C, and plasma was separated. Plasma samples were labeled and maintained frozen at −20 °C till analysis.

#### 2.5.4. Pharmacokinetic analysis

All the required pharmacokinetic parameters were calculated from plasma concentration–time data of MSP by non-compartmental pharmacokinetic analysis using Phoenix/WinNonlin (version 6.4.0.768, Pharsight Corporation, NC). The calculated pharmacokinetic parameters of both reference and test formulations of MSP were subjected to analysis of variance (ANOVA) to test the significance of difference.

## 3. Results and discussion

### 3.1. Preparation and characterization of MSP-PLCP

#### 3.1.1. Determination of the percent yield in prepared MSP-PLCP

The percent yields of MSP-PLCP are shown in Table 1. The percent yield ranged from 60.79% to 96.06%. The results showed that MSP-PLCP prepared from SB2 had a significant higher yield than those prepared from SB1 (*p* < 0.05), while the molar ratio effect on the percent yield was non-significant (*p* > 0.05). Two different types of phospholipid were used in this study. The main phospholipid components are phosphatidylcholine (PC), phosphadylethanolamine (PE), phosphatidylinositol (PI), phosphatidic acid (PA) and other minorities (Ushikubo & Cunha, 2014). To explain the effect of phospholipid type on percent yield, we must first take a look at the composition of two different types of phospholipids used.

According to the manufacturer product information and analysis report (Sigma-Aldrich), SB2 is a crude extract of soybean phospholipids with relatively low PC content (≈19%) and relatively high PI content (≈32%). While SB1 is a pure soybean PC prepared from SB2 by solvent extraction and precipitation procedures, so it has a higher PC content (≈63%) and very low PI content (≈2%). But both types have almost the same PE content (≈30%). Accordingly, the order of PC content is SB1 > SB2, and the order of PI content is SB2 > SB1.

The main interaction pathway between MSP and PL components is weak physical interaction by hydrogen bond formation and electrostatic interaction to form drug–phospholipid complex. This drug–PL complex can form vesicles when dispersed in aqueous medium. This vesicle can be named as complexosomes. The two types used of PL have almost the same PE content but different PC and PI content. By deeply exploring the structural differences between PE, PC, and PI, from Figure S2 in Supplementary material it can be concluded that PI (PubChem CID: 44134894) has six hydroxyl groups, which act as excellent hydrogen bond donating groups, while PC (PubChem CID: 5287971) has eight hydrogen bond accepting atoms and no hydrogen bond donating groups. PE (PubChem CID: 16217018) is a major hydrogen bond accepting molecule with 14hydrogen bond accepting atoms and 2 hydrogen bond donating groups. On the other hand, MSP (PubChem CID: 119584) molecule has six hydrogen bond accepting O or N atoms and has two hydrogen bond donating sites. Citrate moiety adds extra four hydrogen bond donating and seven hydrogen bond accepting groups (PubChem CID: 119583), which can also interact with PL components. Hence, it can be deduced that MSP is major hydrogen bond accepting molecule, PI is a major hydrogen bond donor molecules, PC has no hydrogen bond donating properties and PE is a major hydrogen bond accepting molecule. Accordingly, MSP is more like to strongly interact with PI molecule. Hence, the higher yield% from SB2 than SB1 can be explained by the higher PI content of SB2 compared with SB1 and the higher tendency for hydrogen bond formation between MSP and PI molecule.

#### 3.1.2. Determination of *n*-octanol/water partition coefficient (*P*) of MSP and MSP-PLCP

Increasing MSP partition coefficient is one of the most important outcomes of drug–phospholipid interactions. *n*-Octanol/water partition coefficient (*P*) and log(*P*) of MSP and MSP-PLCP are shown in Table 1. It is obvious that preparation of MSP-PLCP leads to significant increase in partition coefficient compared to pure MSP. The minimum increase in partition coefficient was in (CP 2) with less than 2-fold increase in *P* relative to MSP. The maximum increase in partition coefficient was in (CP 3) with about 12-fold increase in *P* relative to MSP. Statistical analysis of the results showed that the phospholipid type and molar ratio had a significant effect on *n*-octanol/water partition coefficient (*P*) of MSP-PLCP (*p* < 0.05).

The results showed that MSP-PLCP prepared using SB2 had higher partition coefficient than those prepared using SB1. It is well known that the PI enriched fraction of PLs is a good water-in-oil emulsifier with higher overall lipophilic characters with a lower HLB than the PC or PE-enriched fractions, which is considered as an excellent hydrophilic oil-in-water emulsifier with a higher HLB (Van Nieuwenhuyzen, 1981; Hammond et al., 2005; Cabezas et al., 2016). SB2 has the highest PI content (about 32%), so MSP-PLCP prepared using SB2 had a higher partition coefficient and lipophilic character. SB1, having almost no content of PI, produced MSP-PLCP with lower partition coefficient and lipophilic character. It can be concluded that formation of a complex with PI component of PL lead to a much more increase in partition coefficient than formation of PL-complex with PC component.

The results showed also that increasing the molar ratio from 1:1 to 1:2 significantly increased partition coefficient. This increase can be attributed to higher amount of PL relative to the amount of the drug.

#### 3.1.3. Solubility studies in water and *n*-octanol

Solubility studies in water and *n*-octanol were performed to monitor the change in both hydrophilic and lipophilic characters of the prepared MSP-PLCP. Results of these solubility studies are presented in Table 1. Statistical analysis of the results showed that the phospholipid type and molar ratio have a significant effect on water solubility of MSP-PLCP (*p* < 0.05), while only phospholipid type had a significant effect on *n*-octanol solubility of MSP-PLCP (*p* < 0.05).

Formation of MSP-PLCP leads to a decrease in water solubility and an increase of *n*-octanol solubility of MSP, that is, decrease in the hydrophilic character and increase in the lipophilic character. The decrease in water solubility of MSP-PLCP could be due to the sticky nature of the formed complex, which may retard dissolution (Jiang et al., 2016). On the other hand, the increase of *n*-octanol solubility was expected as PL increased lipophilic character of the formed complex. The significant effect of molar ratio on water solubility could be only noticed in MSP-PLCPs prepared by SB2. As mentioned before, PI is a lipophilic molecule, which is found in high proportions in SB2, so upon doubling SB2 amount, the increase in PI content relative to the drug lead to a significant decrease in water solubility.

#### 3.1.4. Measurement of *in vitro* SFRT

Increasing the mucoadhesive properties of the drug molecules will increase the residence time of the drug particles on various mucosal surfaces, hence increasing its absorption. *In vitro* SFRT of MSP and the prepared MSP-PLCP are listed in Table 1. Results showed that the prepared MSP-PLCP have longer *in vitro* mucoadhesion time than MSP. Statistical analysis of the results showed that that the phospholipid type and molar ratio have a significant effect on *in vitro* mucoadhesion time of MSP-PLCP (*p* < 0.05).

Regarding soybean lecithin, results showed that SB1 formulae had higher SFRT than SB2 formulae. This behavior may be linked to *n*-octanol solubility. *n*-Octanol solubility is an indication for the hydrophobic character of the molecule. The mucus gel consists of mucin glycoproteins, lipids, inorganic salts, and up to 83% water (Schäfer-Korting, 2010). The first step involved in the formation bioadhesive bonds is wetting and swelling of the bioadhesive molecule to permit intimate contact with biological tissue (Mathiowitz et al., 2013). Therefore, as hydrophobicity increases, the wetting and swelling step will be hindered, and mucoadhesive properties will decrease. This could explain the results of SFRT. SB1 formulae had lower *n*-octanol solubility (hydrophobic character) and higher SFRT when compared to SB2 formulae. However, it is worth mentioning that this hydrophobic character can be considered as an advantage if the formed complex passed inside the gastrointestinal tract. The stomach lining is relatively hydrophobic due to the presence of a hydrophobic phospholipid lining on the top of the luminal surfaces of gastric mucosa (Hills, 1996). Unlike other hydrophilic mucosal surfaces, the hydrophobic stomach surface is essential to protect the mucosal surface against damaging factors such as acids, mechanical erosion, and digestive enzymes. This hydrophobic phospholipid lining of the mammalian gastric mucosa acts as an adhesion barrier to hydrophilic bioadhesive polymers, nevertheless, adhesion is improved when the surface hydrophobicity of polymers increases (Park & Robinson, 2008). On the other hand, results showed that increasing molar ratio lead to a significant increase in SFRT, which was expected due to the increase in phospholipid content.

#### 3.1.5. PS analysis of MSP-PLCP

When the particles are of nanometer length scale, they can have deep access to the human body because of the PS and control of surface properties (Gupta & Kompella, 2006). The nano-sized particles also show a strong tissue adhesion because of the increased contact area for bioadhesive interactions (Lamprecht et al., 2001; Chow, 2003). The average PS of the prepared MSP-PLCP is listed in Table 1. Results showed that all the prepared MSP-PLCP are in the nanometer length scale. Statistical analysis of the results showed that molar ratio had a significant effect on PS of MSP-PLCP (*p* < 0.05), while the phospholipid type effect was non-significant (*p* > 0.05). It is known that Soybean lecithin (vegetable source) contains high percent of unsaturated fatty acids. Recently, it was found that vesicle stability is a function of fatty acid unsaturation degree. As the degree of unsaturation increases, the membrane becomes more fluid like and membrane thickness is reduced. As a consequence the membrane will become more flexible and the PS of the vesicles is expected to be smaller (Teo et al., 2011). This may explain the significant decrease in particle size with increasing PL content.

#### 3.1.6. Transmission electron microscopy

**Figure 2.**
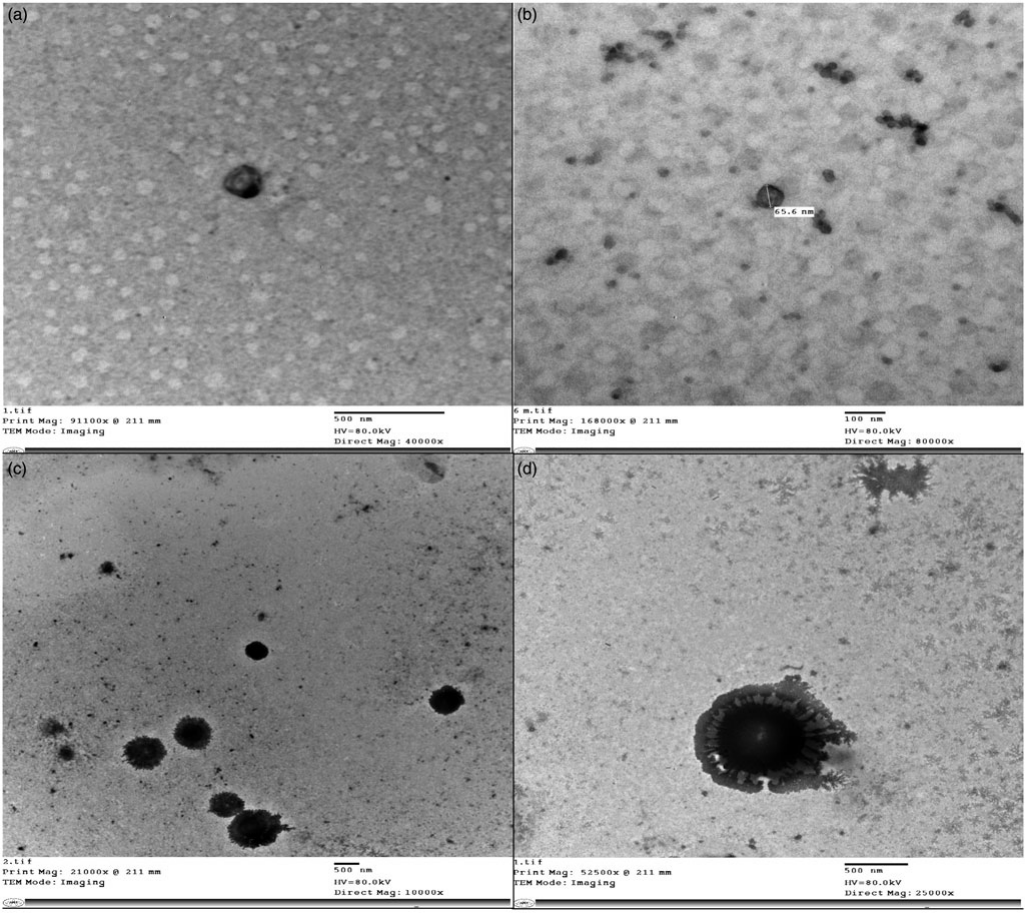
TEM micrographs of optimum MSP-PLCP (a and b) and TEM micrographs of the optimized tablet dispersion (c and d) in simulated saliva.

TEM micrographs of optimum MSP-PLCP (CP 4) after dispersion in distilled water are shown in Figure 2(a,b). It can be seen that MSP-PLCP formed a vesicular structure in aqueous dispersions. The formed vesicles or complexosomes are small with thin walls. These findings are consistent with results and explanations of PS measurements.

#### 3.1.7. Fourier-transform infrared spectroscopy

The FT-IR spectra for MSP, physical mixtures of MSP and phospholipids and the prepared MSP-PLCP are all included in Supplementary material.

The FT-IR spectrum of MSP (Figure S3) shows absorption bands at 3444 cm^−1^ and 3379 cm^−1^ belonging to the N–H primary stretching vibration (Ali & Sayed, 2013). Another absorption band at 3332 cm^−1^ belongs to the secondary –NH in monosubstituted amide (–CONH) stretching vibration (Ali & Sayed, 2013). An absorption band at 3230 cm^−1^ belongs to the hydroxyl groups (–OH) from citric acid (Fujita, 1982). A strong absorption band at 1724 cm^−1^ corresponds to citric acid carbonyl groups stretching vibrations (Fujita, 1982). A characteristic absorption band at 1635 cm^−1^ corresponding to the carbonyl amido (–CONH) stretching vibration. This lies in the lower range for carbonyl group absorption. It is known that conjugation of a carbonyl with a phenyl group typically lowers the carbonyl absorption frequency (Larkin, 2011). NH_2_ deformation band can be observed at 1622 cm^−1^. A strong band at 1546 cm^−1^ involving the CNH bend and the C–N stretching vibrations.

In the spectra of the physical mixtures (Figures S4 and S5), the aforementioned characteristic absorption peaks of MSP are still present. However, the IR spectra of MSP-PLCP were significantly different where some of the characteristic absorption peaks of MSP are masked and new peaks were observed (Figures S6–S9). The characteristic absorption peaks from 3200 to 3500 cm^−1^ were masked in the IR spectrum of MSP-PLCP. This suggests that some weak physical interactions between MSP and phospholipids (mainly PC) took place leading to the formation of drug phospholipid complex. An overlapping between the amide C = O stretch band and the NH_2_ deformation can be observed in MSP-PLCP IR spectra. This overlapping between these two peaks indicates a state of hydrogen bonding (Lin-Vien et al., 1991). Citric acid carbonyl group absorption band was shifted from 1724 cm^−1^ to a higher frequency range (1730–1750 cm^−1^). This can be taken as an evidence for possible complexation between citric acid carboxylic groups and phospholipids. It has been reported that any drug with an active hydrogen atom (–COOH, –OH, –NH_2_) can be esterified to the lipid with or without a spacer arm (Semalty et al., 2009). MSP have no carboxylic group but have another active hydrogen atom –NH_2_, which is involved in salt formation with citrate moiety and can be esterified indirectly using citrate as a spacer arm. This esterification reaction can be self-catalyzed homogeneously by mono- and di-citrate esters (Kolah et al., 2006). But, it is important to state that this assumption cannot be confirmed by the findings of this study and needs more extensive investigations to be proven using pure PL components (PC, PI, and PE) and citrate in tetrahydrofuran to identify type and nature of this interaction.

#### 3.1.8. Differential scanning calorimetry

Figures S10 and S11 in Supplementary material represent DSC thermograms performed for MSP, physical mixtures of MSP and phospholipids and the prepared MSP-PLCP. The thermogram of MSP showed a characteristic endothermic peak at 113 °C that corresponds to the melting point of the drug (ElMeshad & El Hagrasy, 2011; Kim et al., 2011). This characteristic peak could still be detected in the physical mixtures’ thermograms. DSC thermograms of MSP-PLCP show the disappearance of this endothermal peak. This strongly suggests the occurrence of interactions between MSP and PL.

#### 3.1.9. Powder X-ray diffraction

XRD patterns performed for MSP, physical mixtures of MSP and phospholipids and the prepared MSP-PLCP are all included in Supplementary material (Figures S12 and S13). MSP powder diffraction pattern displayed sharp crystalline peaks, which is the characteristic of a crystalline macromolecule. In the physical mixture, crystalline drug signal was still detectable. However, the crystalline peaks had disappeared in MSP-PLCP. This suggested that MSP-PLCP was in amorphous (Cui et al., 2006).

#### 3.1.10. Selection of MSP-PLCP optimal formulation through the determination of the desirability factor

According to the desirability factor, CP4 which had the highest desirability factor (0.74) was selected for further formulation.

### 3.2. Preparation and characterization of SFRP containing the optimum MSP-PLCP formula

#### 3.2.1. Disintegration and wetting time

*In vitro* disintegration and wetting times of the prepared SFRPs are represented in Table 2. It can be found from the results that all the prepared SFRPs had a disintegration time ranged from 3 to 27 s. These results are within the pharmacopeial limits for fast disintegrating SFRPs, which is 3 min. An upper limit for the wetting time was set to 3 min. If no complete wetting occurs within 3 min, the wetting time is considered to be 180 s.

The quadratic equations that describe the quantitative effects of the independent variables on the disintegration and wetting time responses are listed in Table S1 in Supplementary material. ANOVA analysis of the data indicated that only *A* (polymer concentration) and *A*^2^ were significant model terms (*p* < 0.05). From Table 2, it can be noticed that *R*^2^, adjusted *R*^2^, and predicted *R*^2^ are close to the unity, which indicated that the model was statistically excellent. Signal adequacy was indicated by adequacy/precision ratio of 13.5 and 235 for disintegration and wetting time, respectively.

**Figure 3.**
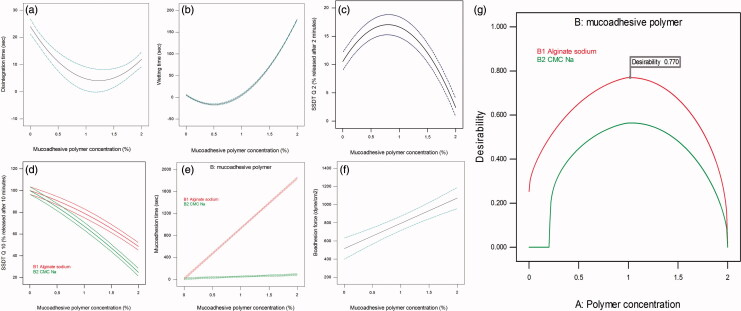
The effect of mucoadhesive polymer concentration on (a) disintegration time, (b) wetting time, (c) SSDT Q2 and (f) bioadhesion force. Combined effect of mucoadhesive polymer concentration and type on (d) SSDT Q10 and (e) mucoadhesion time. Desirability curve for optimization (g).

As implied by the quadratic equation regression coefficients in Table S1, disintegration time and wetting decreased by increasing polymer concentration and increased by increasing the square of polymer concentration. The opposite signs indicated that the mucoadhesive polymer behaved differently in the low concentration and in the high concentration states. From Figure 3(a,b), it can be seen that increasing polymer concentration to about 1% lead to an initial decrease in both disintegration and wetting time. On the other hand, further increase in polymer concentration lead to an increase in disintegration and wetting time. It is important to emphasize that the effect of Na alginate and Na CMC on SFRP properties is dependent on the amount incorporated in the formulation. They both can be used as a tablet disintegrant in low concentrations and can be used in the preparation of sustained release tablets in high concentration (Khan & Rhodes, 1975; Stockwell et al., 1986; Guo et al., 1998; Bi et al., 1999; Bhardwaj et al., 2000; Tønnesen & Karlsen, 2002). In this study, addition of low concentrations of these polymers kept relatively a fast disintegration time.

#### 3.2.2. Determination of *in vitro* SSDT using a novel developed method

This new *in vitro* dissolution method was developed as an attempt to simulate real *in vivo* sublingual conditions and to increase the discrimination ability of any minor changes in the dissolution profile that might occur due to variabilities in the formulation. Results of the *in vitro* SSDT are represented in Table 2.

According to Table S1 in Supplementary material, a quadratic model equation best-fitted SSDT Q2 results. *A* and *A*^2^ were the significant model terms. Initial increase in percent released of MSP can be observed by increasing polymer concentration till a certain point, followed by a significant decrease in percent released by any further increase (Figure 3(c)). It can be observed also that signs of regression coefficient here are opposite to those in disintegration and wetting quadratic equations. This confirms the conclusion that low mucoadhesive polymer concentration can keep fast wetting, fast disintegration, and consequently a faster release rate.

A quadratic equation described effect of independent variables on SSDT Q10 as stated in Table S1 in Supplementary material. *A*, *B*, *AB*, and *A*^2^ were the significant model terms according to ANOVA analysis. The negative signs of the regression coefficients of *A* indicated that increasing polymer concentration resulted in a significant decrease in SSDT Q10. The negative sign of *A*^2^ coefficient revealed that higher concentrations of polymer resulted in a further significant decrease in SSDT Q10. Figure 3(d) shows that Na CMC SFRPs had significantly a lower SSDT Q10 that Na alginate SFRPs. This may be the impact of using a smaller dissolution volume in this novel method. During this test, 3 ml was used for dissolution and replaced every 2 min to simulate the stimulated salivary flow rate, which is about 1.5 mL/min (De Almeida et al., 2008). To more understand this behavior, viscosity of the dispersed SFRP in simulated saliva (η_d_) measured during calculation of bioadhesion force was checked. It was found that η_d_ of 2% Na CMC SFRPs was about 35.75 ± 0.70 Cp, while η_d_ of 2% Na alginate SFRPs was about 26.13 ± 0.17 Cp. Hence, it can be deduced that the higher viscosity of dispersed 2% Na CMC SFRPs retarded MSP release rate. This also can be attributed to the water solubility of two polymers used. Na CMC is dispersed easily in water while Na alginate dissolves slowly (Rowe RC et al., 2006). Na CMC is dispersed easily in water giving high initial drug release rate, but after dispersion it forms a viscous liquid, which leads to a decrease in release rate after time. On the other hand, Na alginate dissolves slowly in water resulting initially in a slower release rate, but after dissolving, it gives a solution which had lower viscosity than that of Na CMC, which lead finally to a higher release rate. The effect of both polymer solubility and viscosity is diminished in USP dissolution test by the large dissolution medium volume (900 ml).

From the above results, it can be concluded that the newly developed dissolution method had a higher resolution power as it can differentiate the effect of minor formulation changes when designing a sublingual drug delivery system. During this study, the newly developed method revealed that that Na alginate SFRPs had initially a slow release rate, which is then gradually increased by time. This can be considered as an advantage in the sublingual delivery system as releasing large initial amount of the drug can increase the chances for salivary washout.

#### 3.2.3. Measurement of *in vitro* SFRT

To resist the flushing action of saliva, it is very important for the SFRP to establish an initial adhesion to the sublingual mucosa followed by gradual dispersion of the SFRP components. This test was done to evaluate the initial bioadhesion of the SFRP surface to the sublingual mucosa. Results of the *in vitro* mucoadhesion time of the prepared SFRPs are presented in Table 2. It is obvious that SFRPs prepared using 2% Na alginate SFRPs had the highest mucoadhesion time as the lower SFRP surface remained attached to the mucin/agar surface to about 30 min with gradual dispersion of the upper surface during this time.

As shown in Table S1 in Supplementary material, a linear 2FI model best fitted the relation between independent variables and *in vitro* mucoadhesion time. ANOVA analysis showed that *A*, *B*, and *AB* were the significant model terms. It can be deduced from the equation that, as expected, increasing polymer concentration significantly increased *in vitro* mucoadhesion time. Figure 3(e) shows that Na alginates had significantly longer mucoadhesion time than Na CMC SFRPs. Na CMC and Na alginate are wet adhesives as they are activated by moistening and will have stronger adhesion once activated (Smart, 2005). As aforementioned, once SFRP is immersed in simulated saliva, Na alginate will dissolve slowly and will have enough time to be moistened and to develop a strong interaction with mucin. On the other hand, Na CMC is fast dispersed and will not allow enough time for moistening and strong adhesion to mucin surface.

#### 3.2.4. Measurement of *in vitro* SFRF

After initial adhesion of SFRP surface to the sublingual mucosa, SFRPs’ components gradually disperse in the saliva. If the dispersed particles possess bioadhesive characters, they can resist the flushing action of the saliva. This test was done to estimate the bioadhesion force between the dispersed particles from SFRP dispersion and the sublingual mucosa. Results of this test are represented in Table 2.

As shown in Table S1 in Supplementary material, a linear equation described the effect of independent variables on bioadhesion force, with only polymer concentration (*A*) as a significant term. From Figure 3(f), it can be found that, as expected, increasing polymer concentration lead to a significant increase in bioadhesion force of the dispersed SFRPs.

#### 3.2.5. Optimization and validation of model

The percent deviation listed in Table S1 in Supplementary material was calculated to compare the actual and predicted values for all 10 design points to confirm the practical validity of the models. The obtained polynomial equations were then used for numerical optimization of the prepared SFRPs according to the desirability function using the Design Expert^®^ software (version 10.0.3). Desirability functions were chosen to minimize disintegration time and wetting time and to maximize SSDT Q2, SSDT Q10, *in vitro* SFRT, and SFRF. The resulting overall desirability is shown in Figure 3(g). The solution with the higher desirability (0.77) was chosen as the optimum formulation. The optimum formulation composed of 0.983% of Na alginate. In order to validate the model, the optimum formulation was prepared practically (*n* = 5) and the response was evaluated. All measured values of the optimized formula responses were evaluated to find if they fell within the 95% PIs (prediction intervals) for each of the responses. As shown in Table S2 in Supplementary material, it was found that all measured values fell within their 95% PIs.

Morphology of vesicles after dispersion of optimum SFRP formulation was examined using TEM (Jeol-200 CX, Joel). The SFRP was dispersed in 1 ml simulated saliva and a drop from dispersion was placed in the form of a thin film on a carbon coated copper grid and then viewed and photographed under TEM. TEM micrographs of MSP-PLCP after SFRP dispersion are shown in Figure 2(c,d). It can be seen that MSP-PLCP retained its vesicular structure in SFRP dispersion. It can be noticed also that some SFRP excipients (most probably the mucoadhesive polymer) are adsorbed on formed vesicles.

### 3.3. Pharmacokinetics in healthy volunteers

The calculated mean pharmacokinetic parameters for the market reference product Fluxopride^®^ and the optimized test formula are given in Table 3 and Figure S14 in Supplementary material. The relative bioavailability of optimized test formulation compared to market product Fluxopride^®^ tablets, judged from the *C*_max_, AUC_0–_*_t_* and AUC_0-inf_ was found to be 136.32%, 132.25% and 139.99%, respectively, indicating the superiority of the test formula over the market product. The result of 90% confidence interval confirms also the superiority of the optimized test formulation over the market product as shown in Table S3 in Supplementary material. During *in vivo* testing, volunteers reported the existence of a sticky mass in sublingual area for about 10–15 min from sublingual administration of test formula while reference market product completely dissolved in less than 3 min. It can be deduced from these results that the developed SFRP was successful in significantly increasing the extent of sublingual absorption of MSP with relative bioavailability reaching about 140% (*p* < 0.05). This increase can be attributed to physicochemical modification of MSP molecule by complexation with PI enriched soybean lecithin and formulation of the formed complex into SFRP using Na alginate polymer.

**Table 3.** Summary of the pharmacokinetic parameters of MSP following the sublingual administration of Fluxopride^®^ and the optimized test formulation.

Parameters	Fluxopride^®^	T2
*C*_max_ (ng/ml)	31.31 ± 9.65	42.38 ± 11.33
*T*_max_ (median) (h)	1.125	1.125
MRT (median) (h)	4.70	5.66
AUC_0–10_ (ng h/ml)	103.25 ± 28.28	136.06 ± 41.43
AUC _0–∞_ (ng h/ml)*	117.41 ± 26.70	165.99 ± 50.02
*t*_1/2_ (h)	3.34 ± 1.28	3.85 ± 0.79
*k* (h^−1^)	0.22 ± 0.06	0.18 ± 0.03

* *p* < 0.05 (0.03).

Figure S15 in Supplementary material shows a mechanistic approach for *in situ* formation of SFRP for drug delivery. Because of the relatively small volume of saliva and short residence time, sublingual route was limited for long time to drugs having good water solubility and a suitable lipophilicity (log *P* between 2 and 4). During this study, we managed to change MSP log *P* from 0.76 to about 1.8 by complexation with PL. At the same time, this complex was formulated into novel SFRP. These SFRPs were not adhesive by the classical meaning of very long residence time; however, they extended the residence time of drug molecule from about 3 min in traditional sublingual fast dissolving tablets to about 10–15 min, which increased the sublingual absorption time window, hence increasing the drug bioavailability. This approach can be used for drugs that do not dissolve rapidly in saliva or those with relatively lower permeability and need longer time for permeation through sublingual mucosa. Based on this, this newly developed sublingual mucoadhesive SFRPs offers a very promising and efficient tool to extend the use of sublingual route and widen its applications other than traditional rapid-onset uses.

## 4. Conclusions

This study was conducted to develop a novel and more efficient sublingual drug delivery system using MSP as a model drug. The study had two stages. The first stage was physicochemical modification of MSP by complexation with PI enriched soybean lecithin to enhance its absorption through sublingual mucosa. Practically, PI enriched soybean lecithin is the cheapest and most economic source for PI. The second stage was formulation of the formed complex into a SFRP. Na alginate was used as a powerful mucoadhesive polymer at an optimum concentration, which balanced between the desired mucoadhesive properties and a reasonable release rate of the drug. Pharmacokinetics study in humans proved the superiority of the developed dosage forms over the ordinary sublingual dosage forms. The developed delivery system offers a very promising and efficient tool to extend the use of sublingual route and widen its applications other than traditional rapid-onset uses.

## Supplementary Material

IDRD_Ahmed_et_al_Supplemental_Content.pdf
