# Supplementary material for: Engineering of a novel optimized platform for sublingual delivery with novel characterization tools: in vitro evaluation and in vivo pharmacokinetics study in human
Source: Drug Deliv. 2017 Jun 9;24(1):918–31. doi: 10.1080/10717544.2017.1334719 (PMC8241021; doi:10.1080/10717544.2017.1334719)
Supplement: IDRD_Ahmed_et_al_Supplemental_Content.pdf [file IDRD_A_1334719_SM2515.pdf]

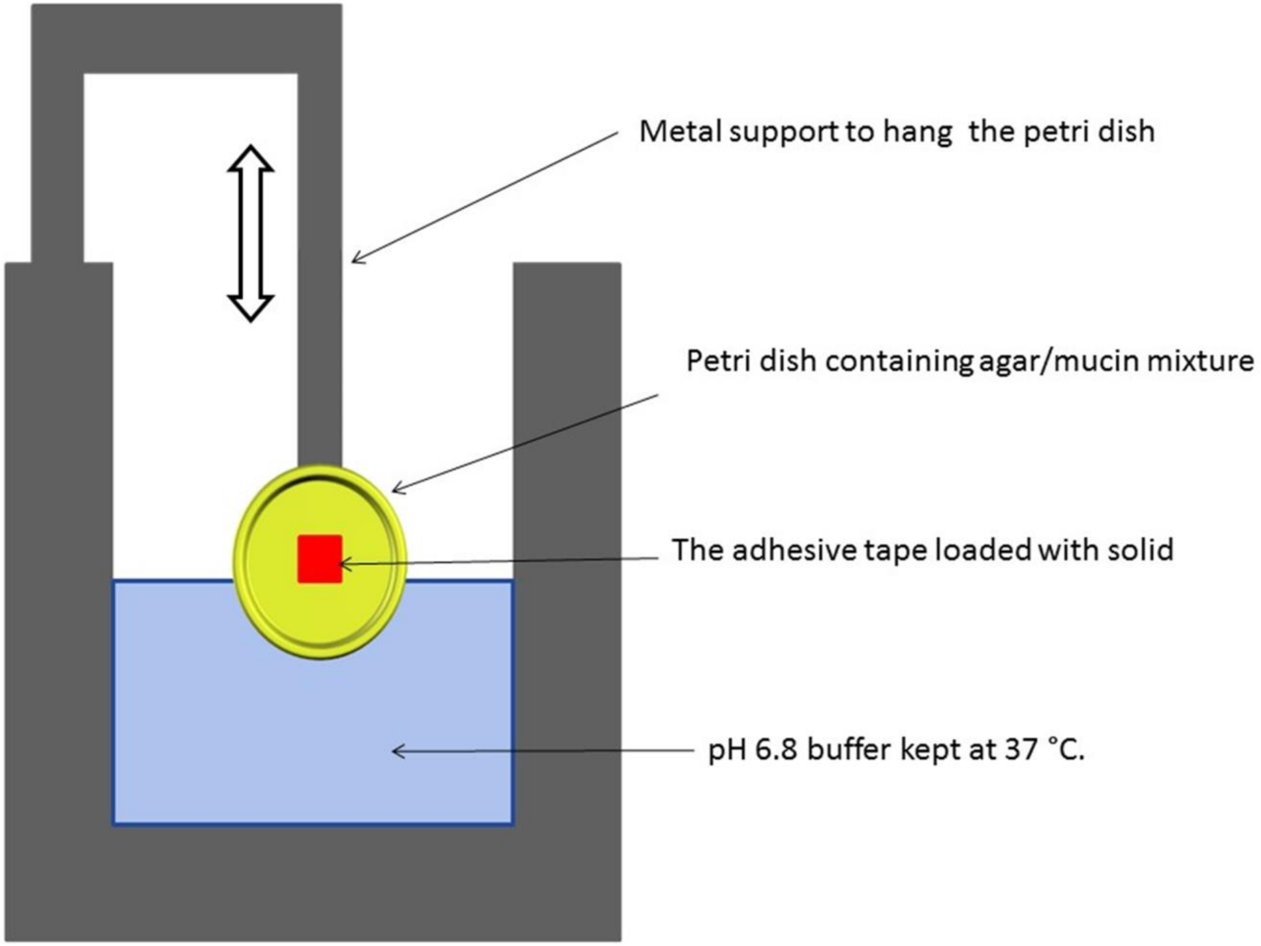

Figure S1: Modified disintegration apparatus for measurement of *in vitro* mucoadhesion time.

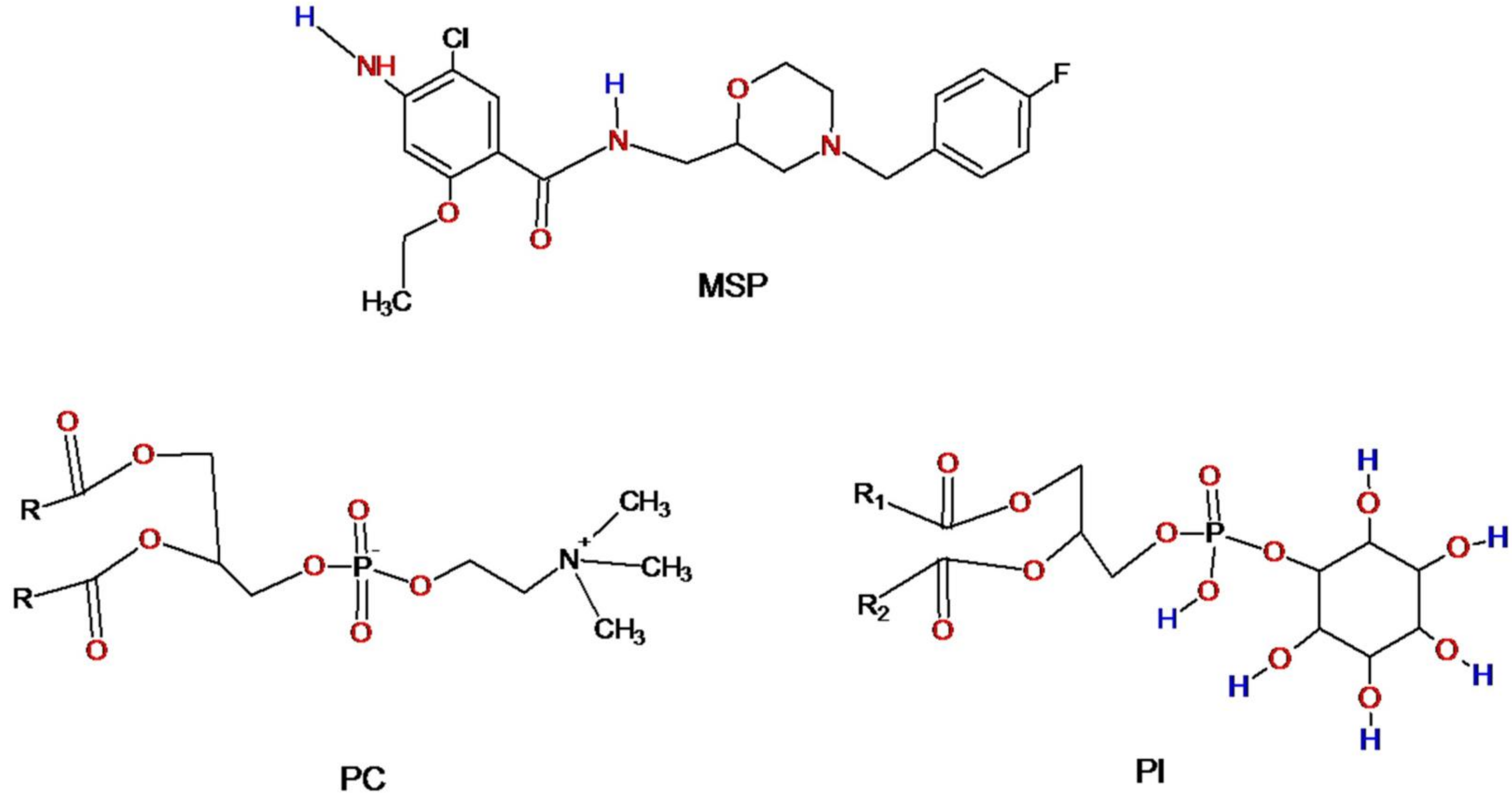

Figure S2: Mosapride (MSP), phosphatidylcholine (PC) and phosphatidylinositol (PI). Blue color indicates hydrogen donating atom or group and red color indicates hydrogen bond receptor atom or group.

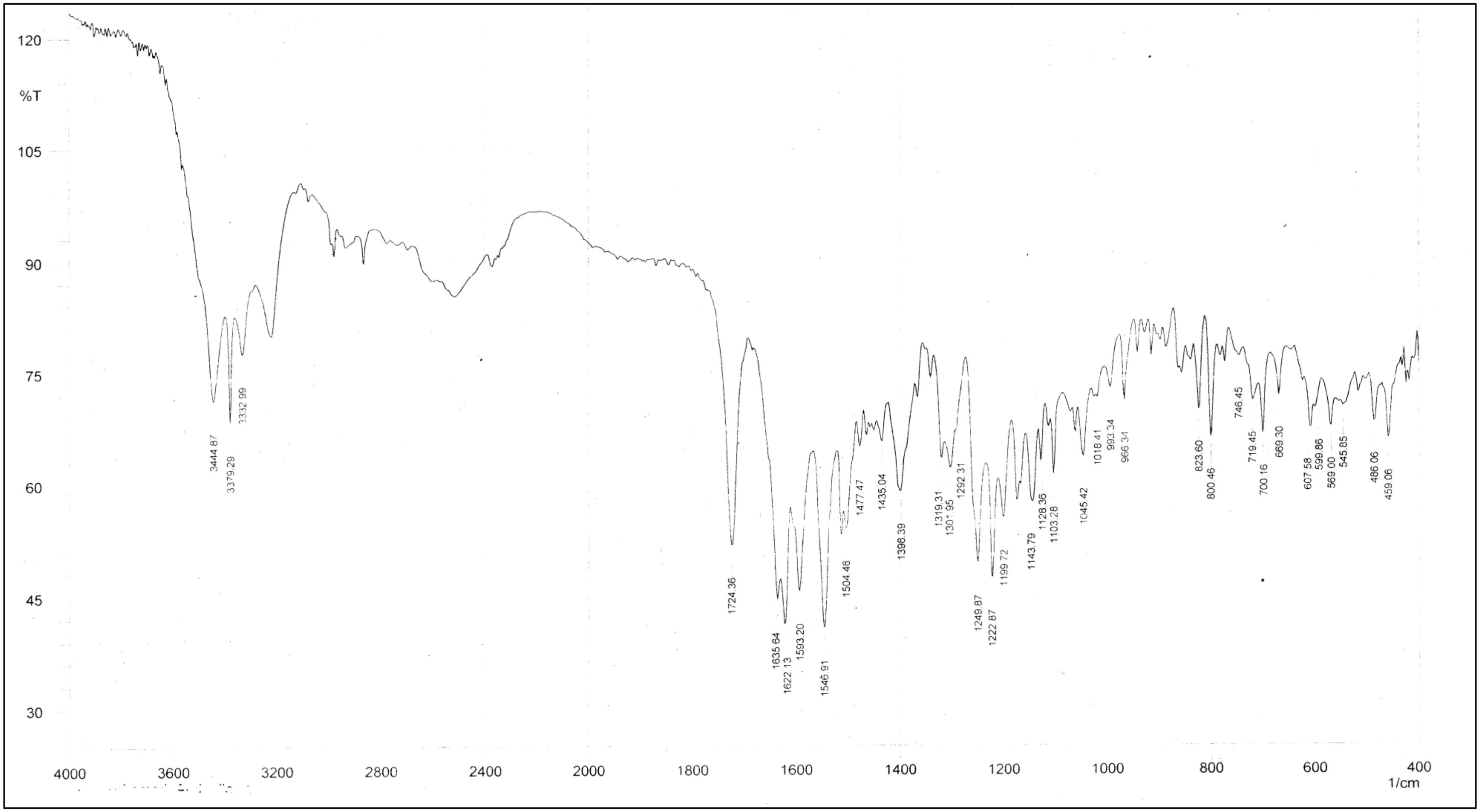

Figure S3. FTIR spectrum of MSP.

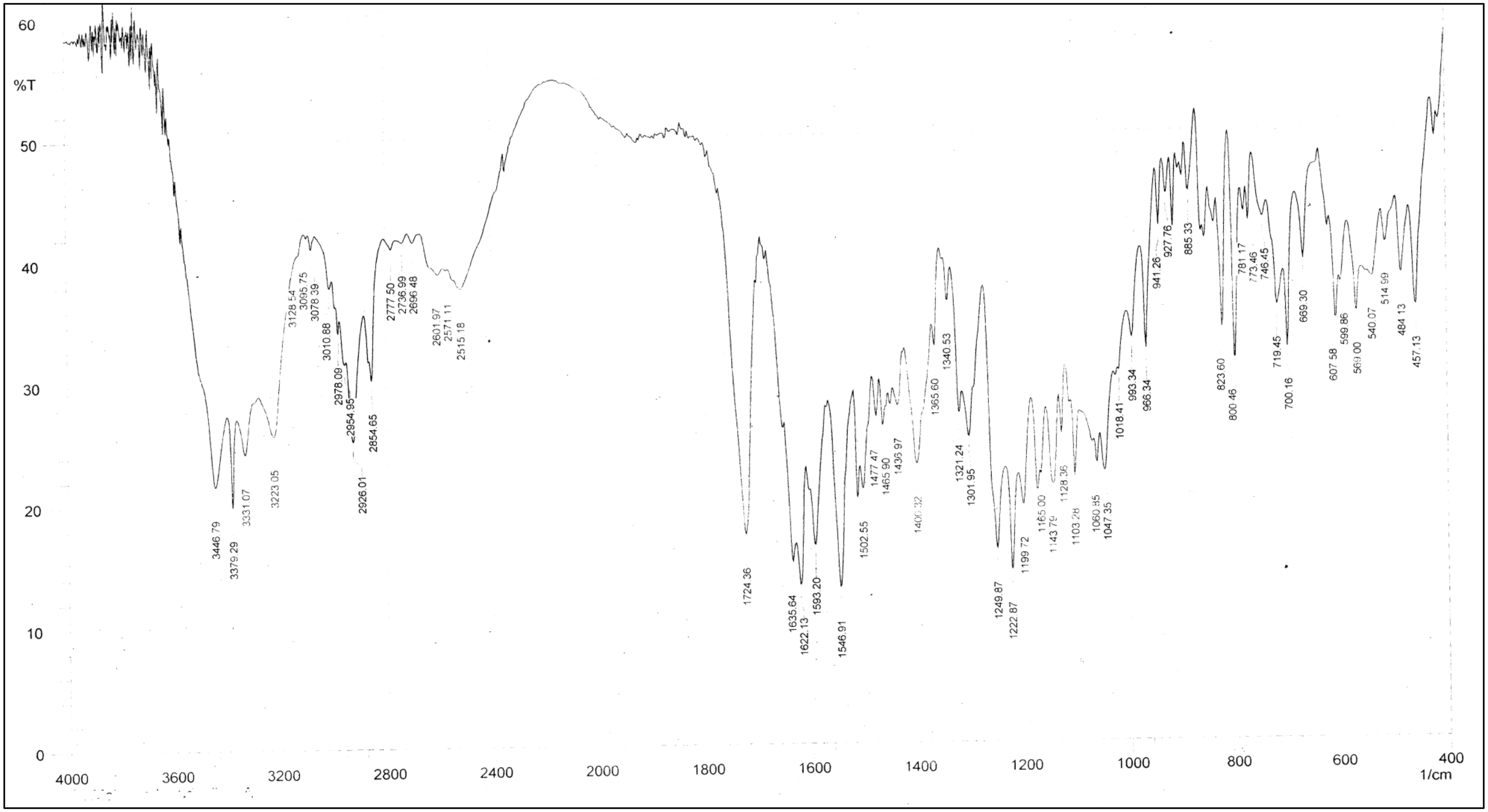

Figure S4. FTIR spectrum of physical mixture of MSP and SB1.

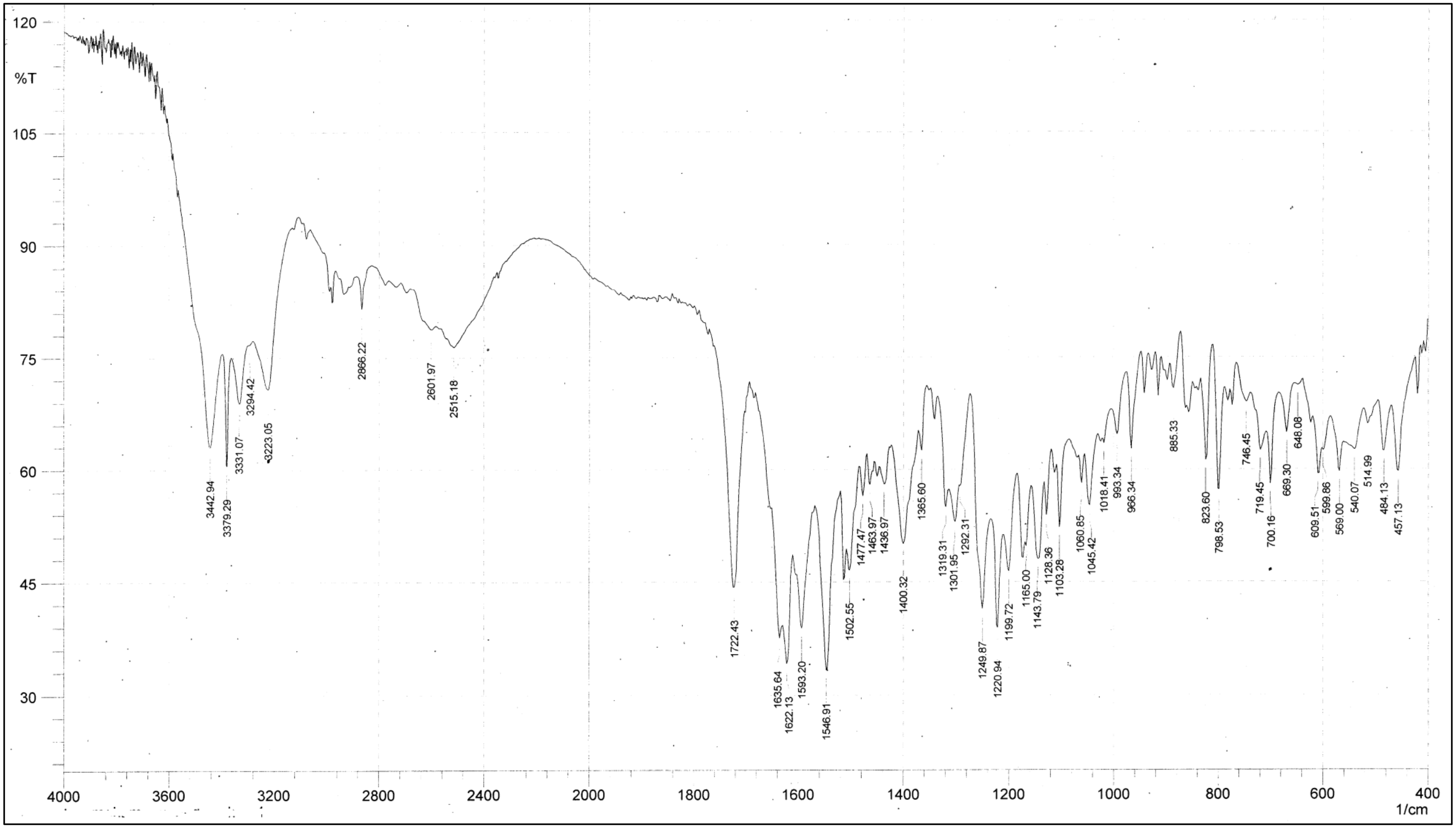

Figure S5. FTIR spectrum of physical mixture of MSP and SB2.

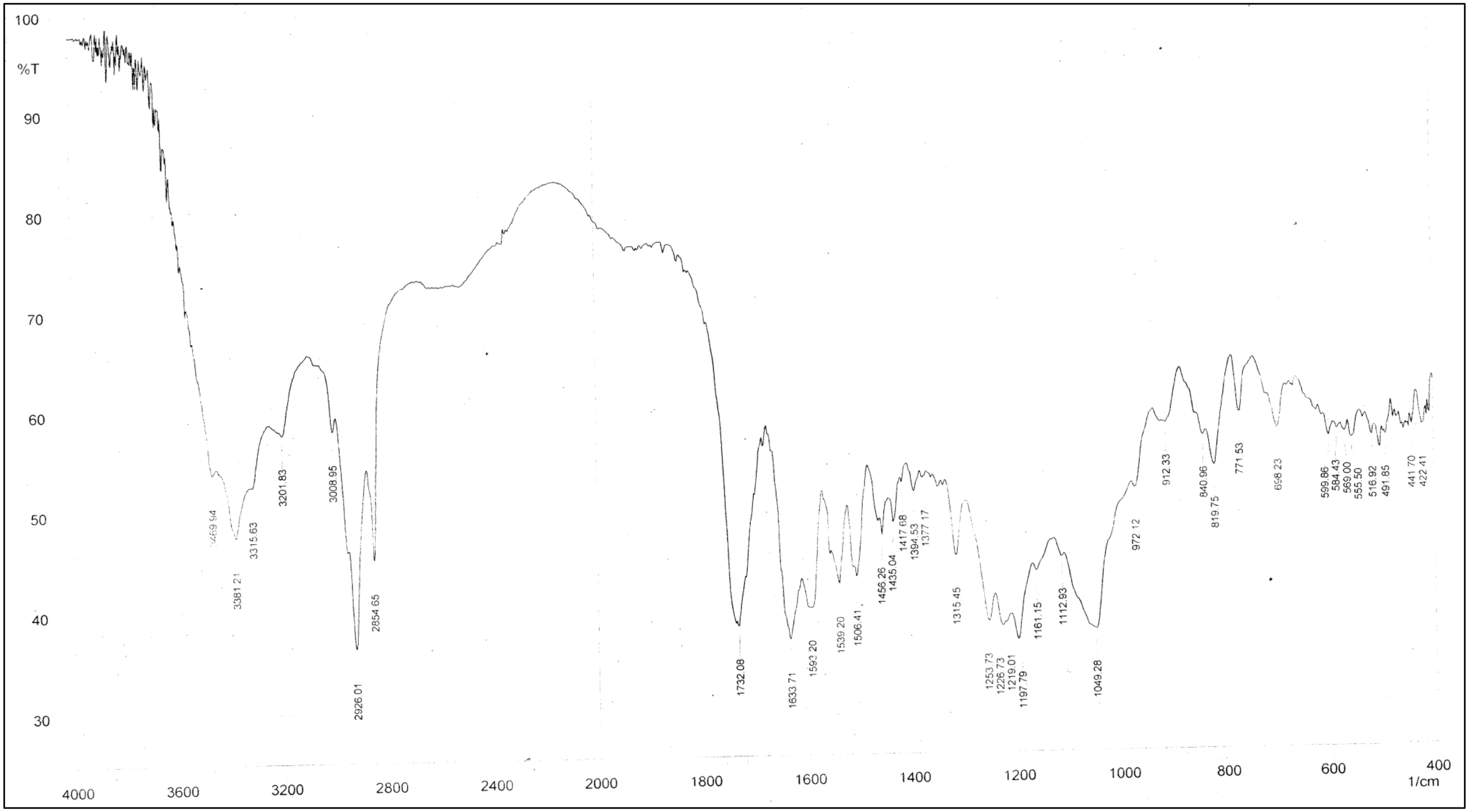

Figure S6. FTIR spectrum of CP1.

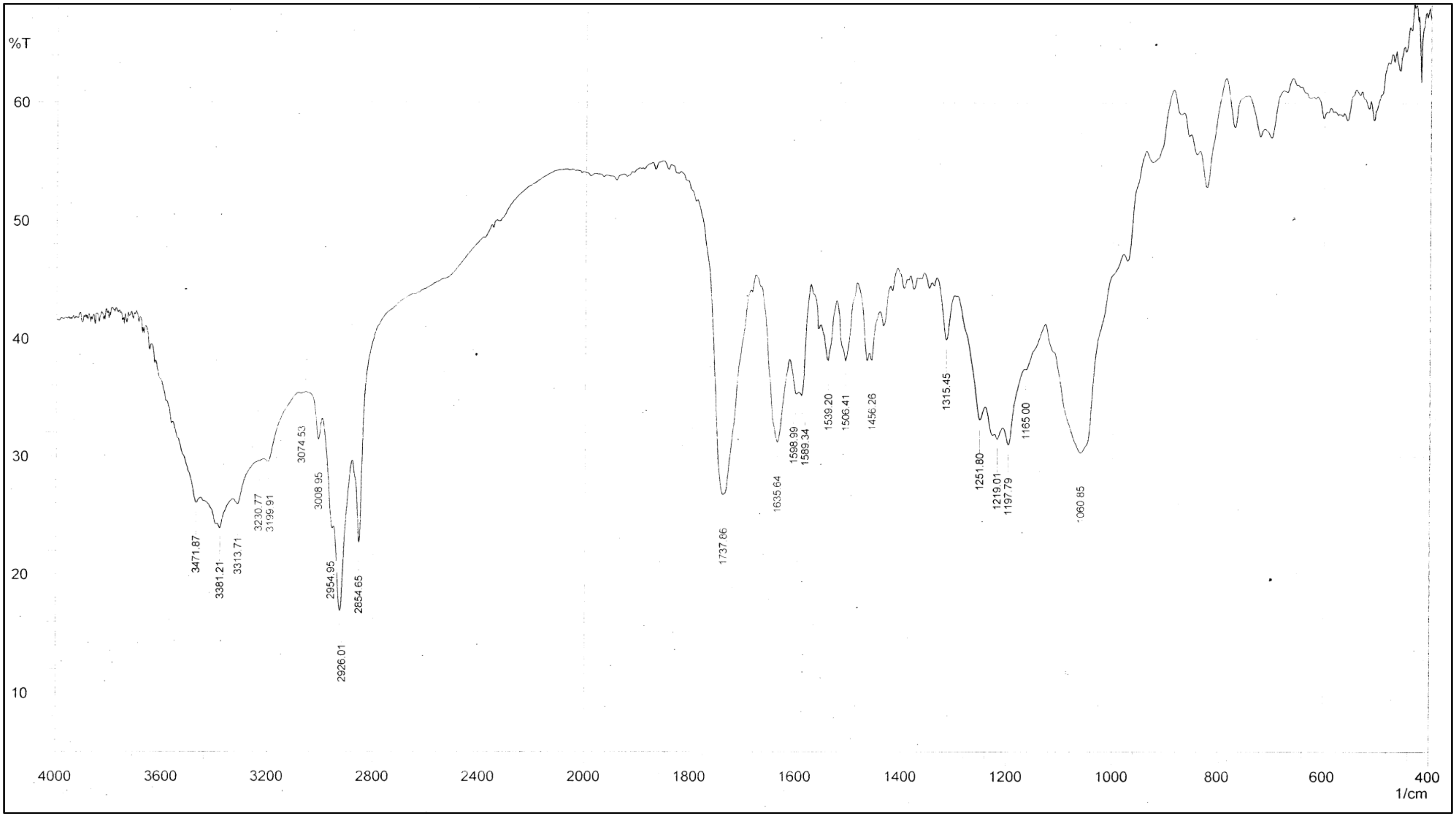

Figure S7. FTIR spectrum of CP2.

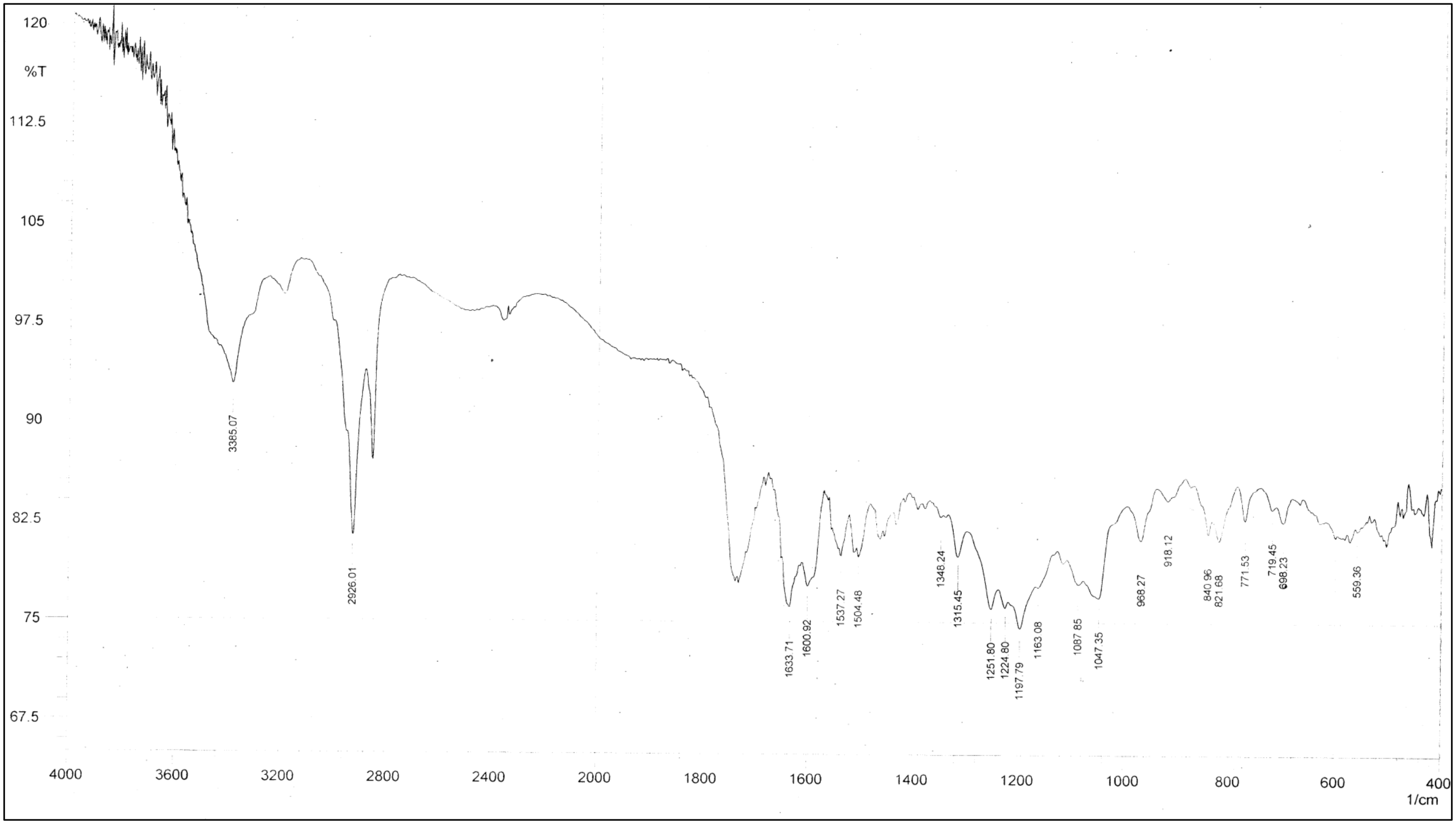

Figure S8. FTIR spectrum of CP3.

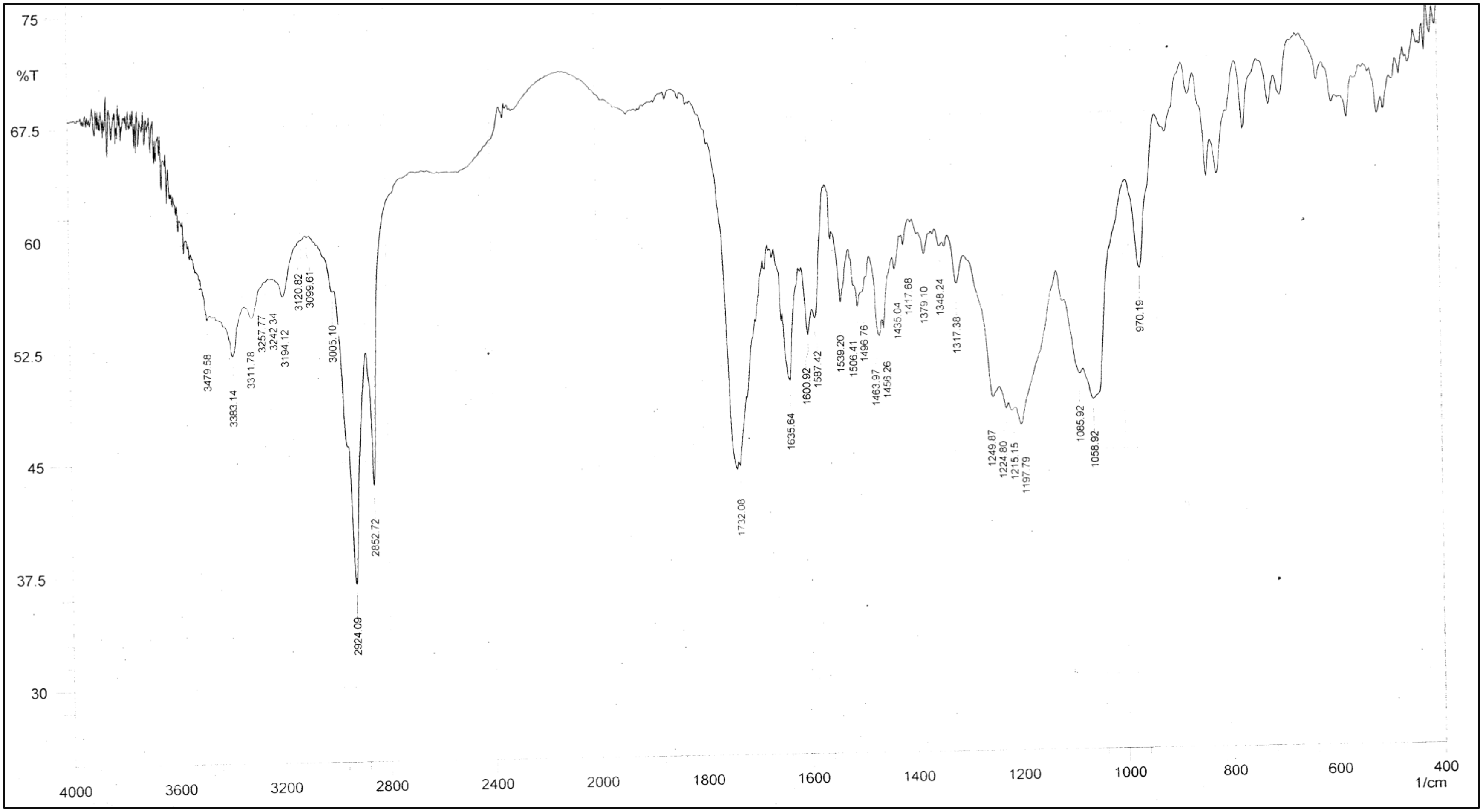

Figure S9. FTIR spectrum of CP4.

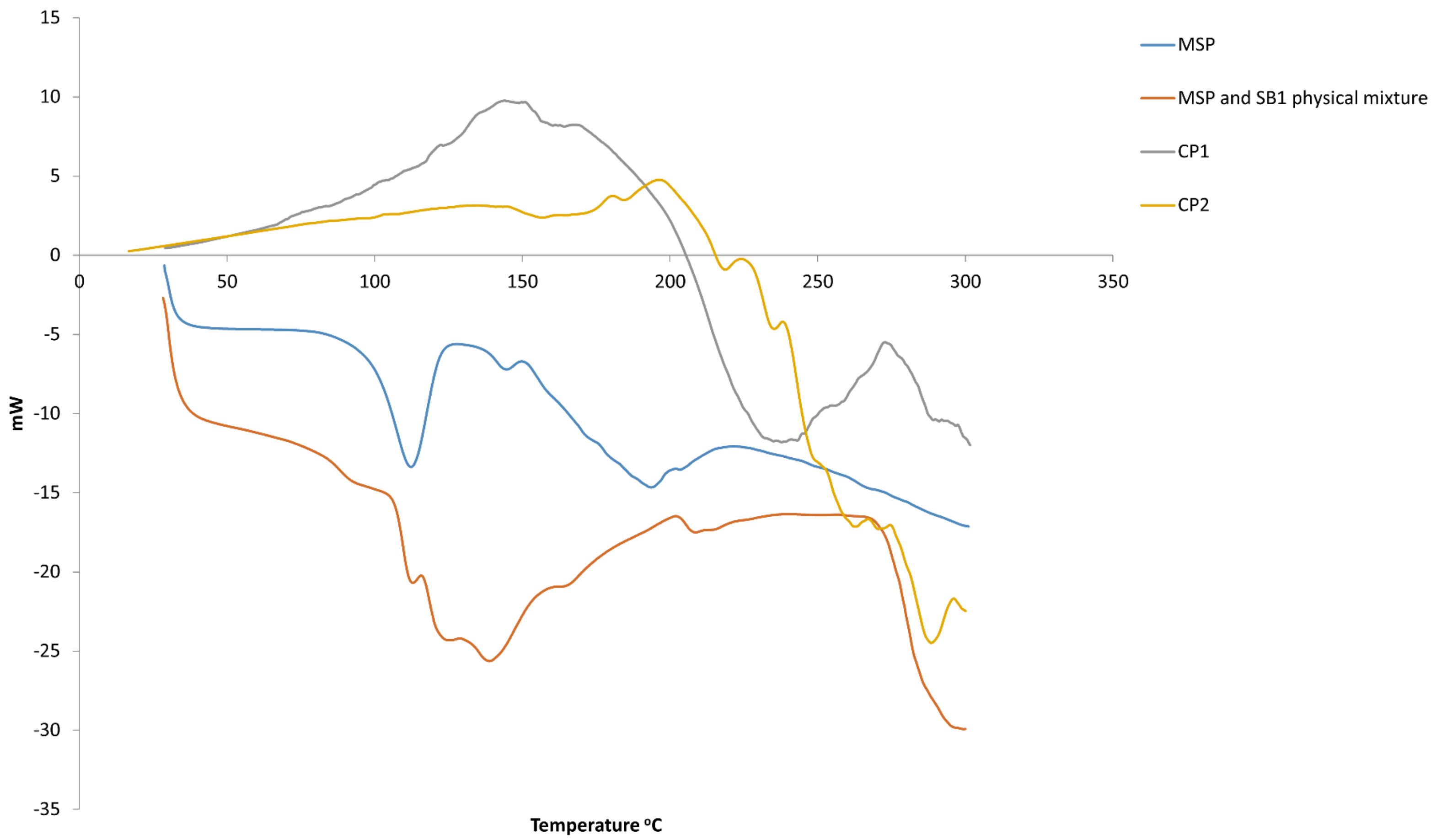

Figure S10. Differential scanning calorimetric (DSC) thermograms of MSP, MSP: SB1 physical mixture, CP1 and CP2.

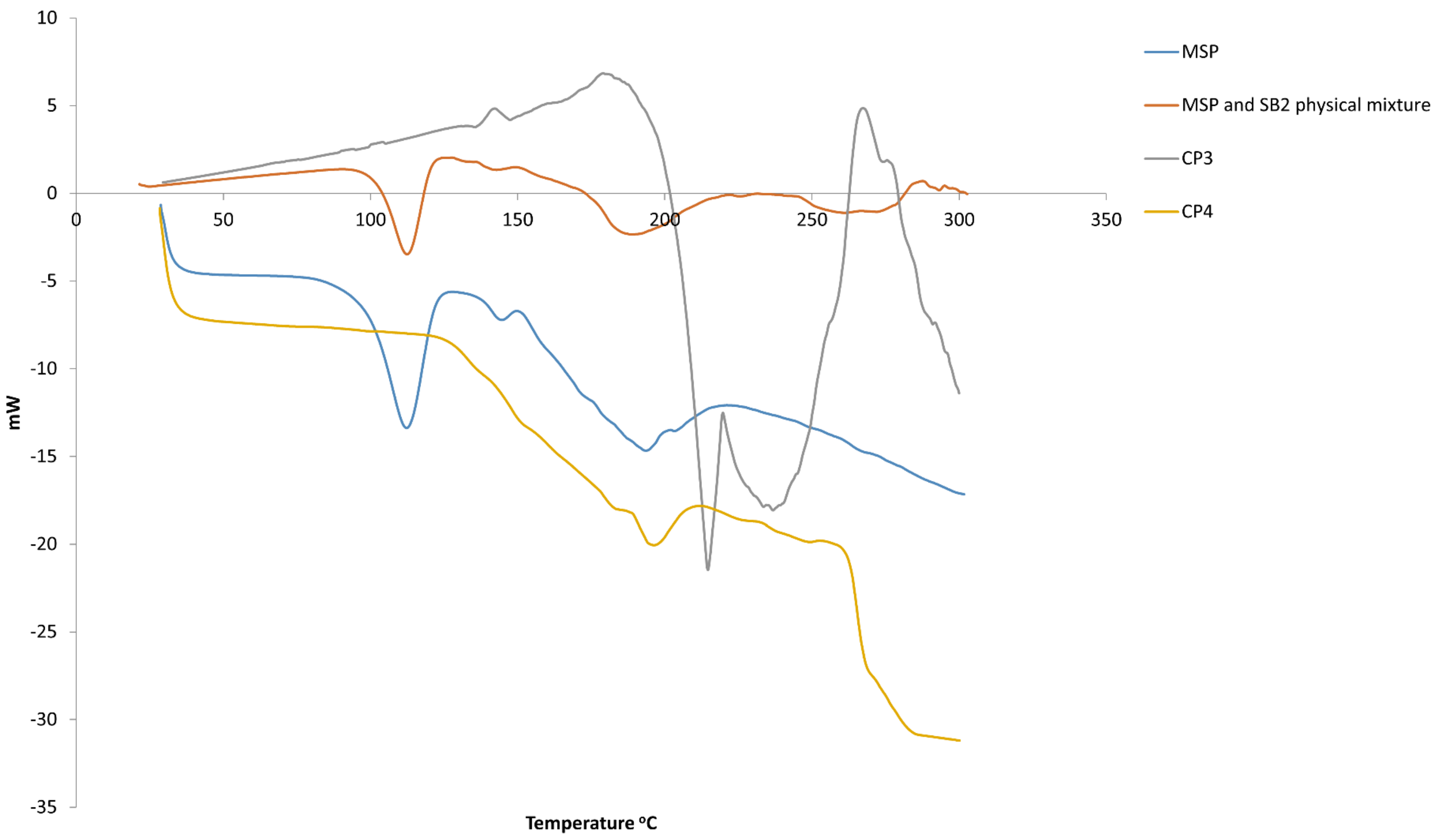

Figure S11. Differential scanning calorimetric (DSC) thermograms of MSP, MSP: SB2 physical mixture, CP3 and CP4.

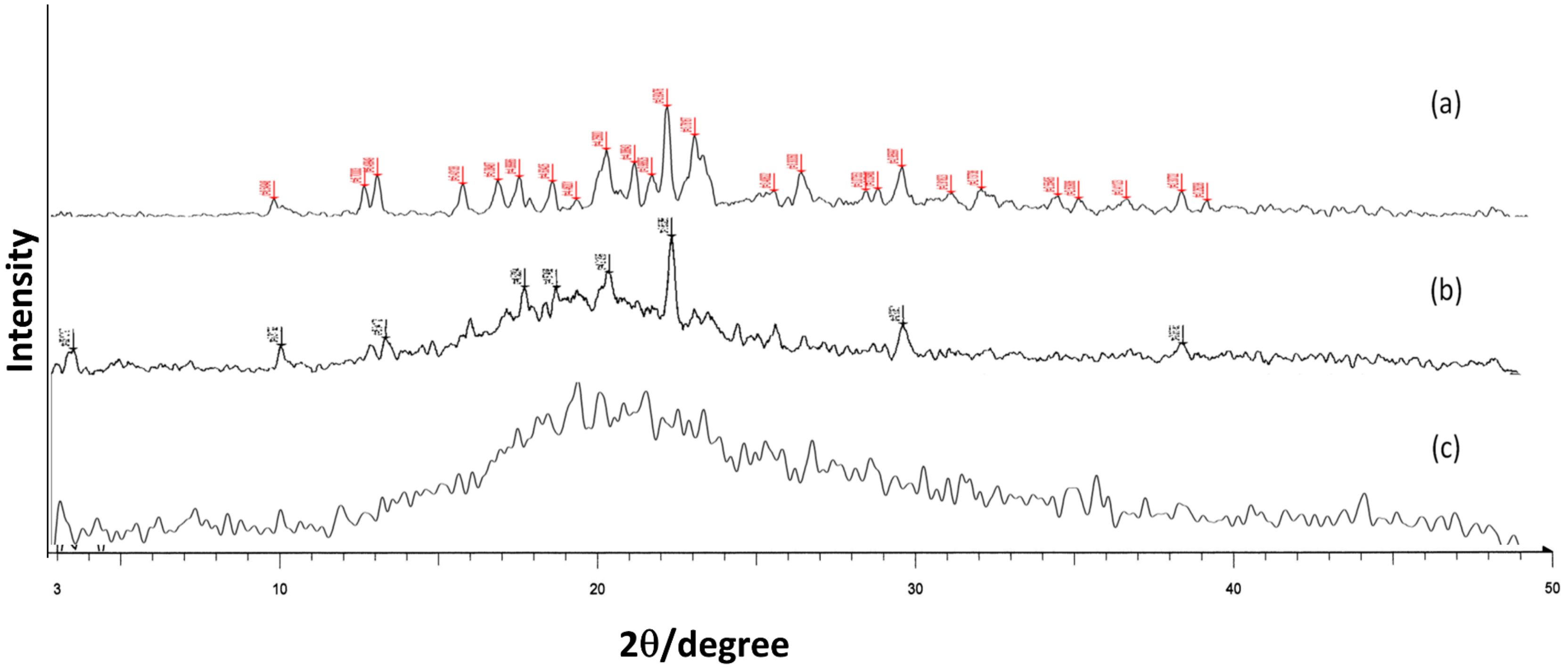

Figure S12. XRD of (a) pure MSP, (b) MSP and SB1 physical mixture, (c) CP1 and (d) CP2.

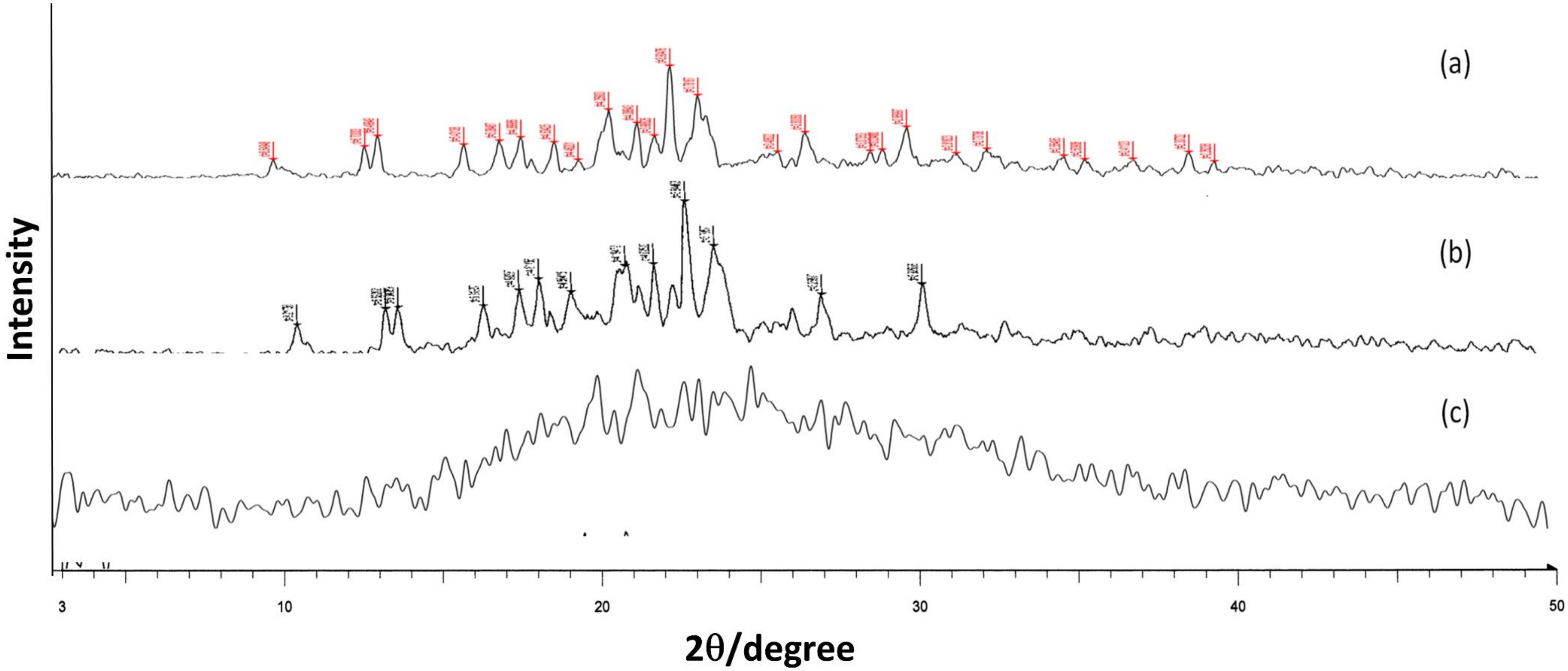

Figure S13. XRD of (a) pure MSP, (b) MSP and SB2 physical mixture, (c) CP3 and (d) CP4.

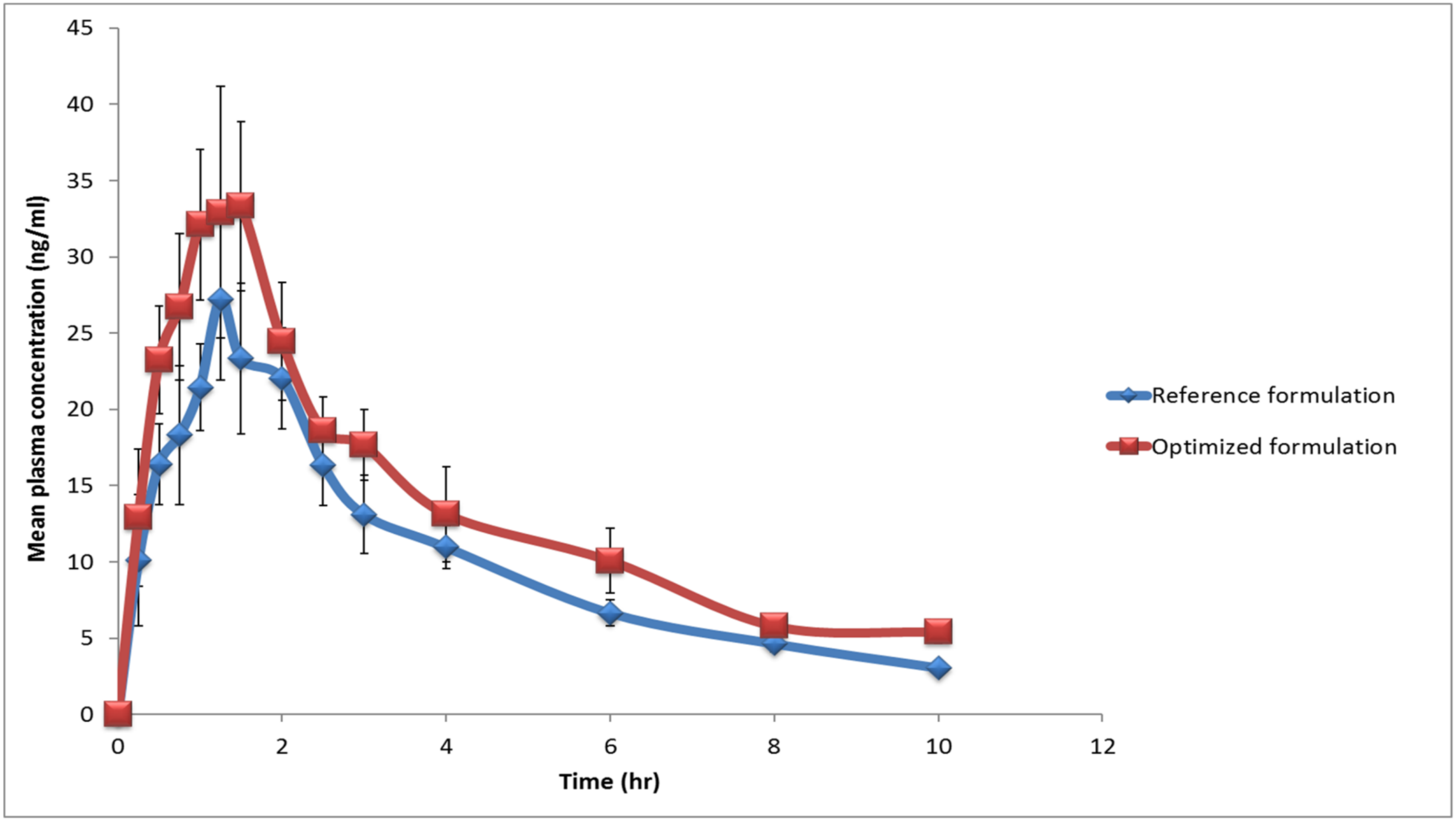

Figure S14: Mean plasma concentration time curve following the sublingual administration of the reference Fluxopride® tablets and the optimized test formulation.

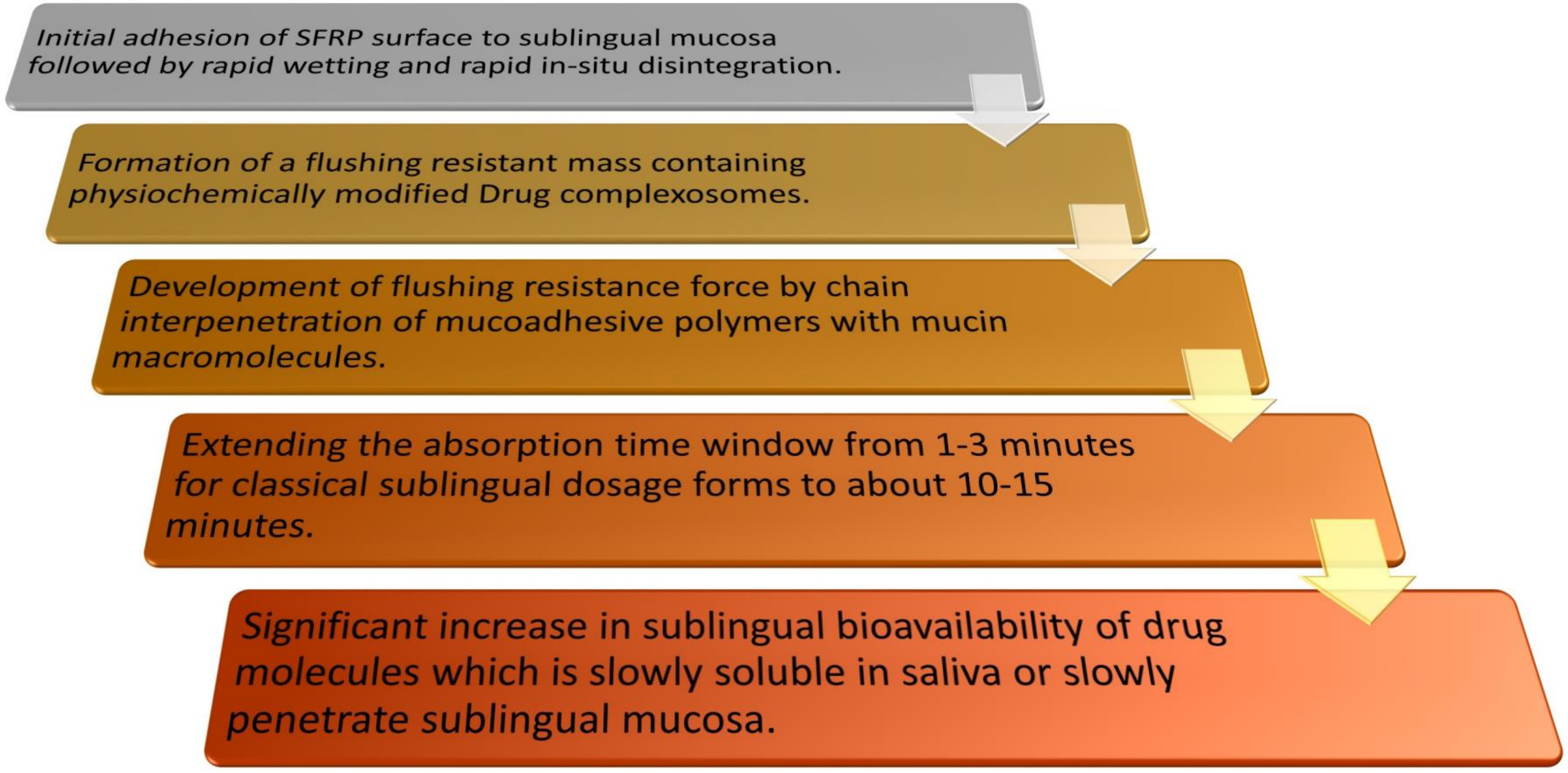

Figure S15: Mechanistic approach for in-situ formation of sublingual flushing resistant platform for drug delivery.

Table S1. Fitted models for the responses, R^2, adjusted R^2, predicted R^2, model equation, significant model terms and average percent deviation of actual results from predicted results.

Response	Model	R^2	Adjusted R^2	Predicted R^2	Adequacy /precision ratio	Model equation*	Significant model terms	Average percent deviation (Average ± SD)
Disintegration time	Quadratic	0.9642	0.9355	0.8566	13.51	$+4.75-6.00\ A-0.75\ B\ -1.25\ AB\ +13.25\ A^2$	A,A^2	14.86 ± 10.45
Wetting time	Quadratic	0.9999	0.9999	0.9997	235.30	$+5.50+87.25A+0.00\ B\ +0.00\ AB\ +87.25\ A^2$	A, A^2	9.56 ± 11.97
USP Q3	Quadratic	0.9981	0.9965	0.9923	51.40	$+97.41-21.43\ A+2.59\ B\ +2.59\ AB\ -18.83\ A^2$	A, B, AB, A^2	1.04 ± 1.35
USP Q10	2FI	0.9710	0.9564	0.9038	18.43	$+98.23-1.83\ A\ +1.54\ B\ +1.48\ AB$	A, B, AB	0.35 ± 0.32
SSDT Q2	Quadratic	0.9949	0.9909	0.9797	36.77	$+16.64-4.07\ A+0.05\ B\ +0.04\ AB\ -10.17\ A^2$	A, A^2	4.94 ± 1.90
SSDT Q10	Quadratic	0.9993	0.9987	0.9971	95.63	$+73.20-31.63\ A-5.95B\ -5.95AB\ -4.82A^2$	A, B, AB, A^2.	1.53 ± 1.97
SFRT	2FI	0.9999	0.9998	0.9996	259.20	$+491.08+476.13\ A-438.92\ B\ -441.88AB$	A, B, AB	16.17 ±18.7
SFRF	Linear	0.8990	0.8701	0.8156	12.15	$+794.58+278.61\ A-64.08\ B$	A	11.59 ±6.32

Table S2: Observed mean, predicted mean and two sided 95 % prediction interval for the optimum formulation.

Response	Observed mean	Predicted mean	Two sided 95 % prediction interval	
			low	high
Disintegration time (sec)	11.5	5.58	-0.45135	11.62572
Wetting time (sec)	5.4	3.99	1.04696	6.952765
Dissolution USP Q 3 (% released)	100	95.23	91.51128	98.95279
Dissolution USP Q 10 (% released)	100	96.75	95.80229	97.69847
Dissolution modified Q 2 (% released)	15.18	16.65	15.102	18.21615
Dissolution modified Q 10 (% released)	80.824	79.59	76.46897	82.72669
SFRT (sec)	906	913.93	895.5502	932.3202
SFRF (dyne/cm^2)	912	853.77	690.4656	1017.093

Table S3:Geometric means, % ratio and 90% confidence intervals for AUCt, AUCinf and Cmax

Pharmacokinetic parameter	Geometric means		% Ratio Test/Ref	90 % Confidence Intervals
	Test	Reference		
AUCt	131.43	99.38	132.25	98.84 – 176.94
AUCinf	160.27	114.49	139.99	112.21 – 174.63
Cmax	41.10	30.15	136.32	106.43 – 174.61
